# Concurrent nanotherapeutics and regulatory updates for the management of amyotrophic lateral sclerosis: a focused review for orphan drug (Tofersen)

**DOI:** 10.1186/s13023-025-04042-2

**Published:** 2025-11-21

**Authors:** Abhiram Kumar, Shivang Shukla, Anjali Rai, Priya Pathak, Kumar Pranav Narayan

**Affiliations:** 1 Laboratory of Molecular Medicine, Department of Biological Sciences, Birla Institute of Technology and Sciences Pilani Hyderabad Campus, Hyderabad, Telangana 500078 India; 2 Department of Pharmacy, Jagannath University, Jaipur, Rajasthan 303901 India; 3 Amity Institute of Pharmacy, Amity University, Lucknow, Uttar Pradesh 226010 India

**Keywords:** Amyotrophic lateral sclerosis, Tofersen, Orphan drug, Regulatory agencies, Pharmacovigilance, Clinical trials

## Abstract

**Background:**

Amyotrophic Lateral Sclerosis (ALS) is a progressive neurodegenerative disorder affecting nerve cells in the brain and spinal cord. With a global incidence of 1.9 to 6 per 100,000 people, ALS is slightly more common in men and prevalent in individuals over 60. However, this review provides a concise update on the regulatory landscape and therapeutic advancements in managing ALS, focusing on the recent approval of Tofersen, the first gene therapy specifically targeting SOD1 mutation-related ALS.

**Results:**

It highlights Tofersen unique role as an orphan drug approved by the US FDA, emphasizing its mechanism of action, gene silencing and its impact on reducing neurodegeneration. Additionally, the review synthesizes data from ongoing clinical trials, pharmacovigilance reports, and case studies to comprehensively understand Tofersen’s safety, efficacy and market exclusivity. Beyond this, it explores the emerging potential of nanotherapeutic approaches to ALS treatment, identifying critical research gaps and future directions.

**Conclusion:**

Integrating regulatory updates, clinical evidence, and innovative therapeutic strategies, the review uniquely contributes to the ALS literature by bridging current treatment realities with potential future therapies, aiming to inform researchers, clinicians, and policymakers on optimizing ALS management.

**Graphical Abstract:**

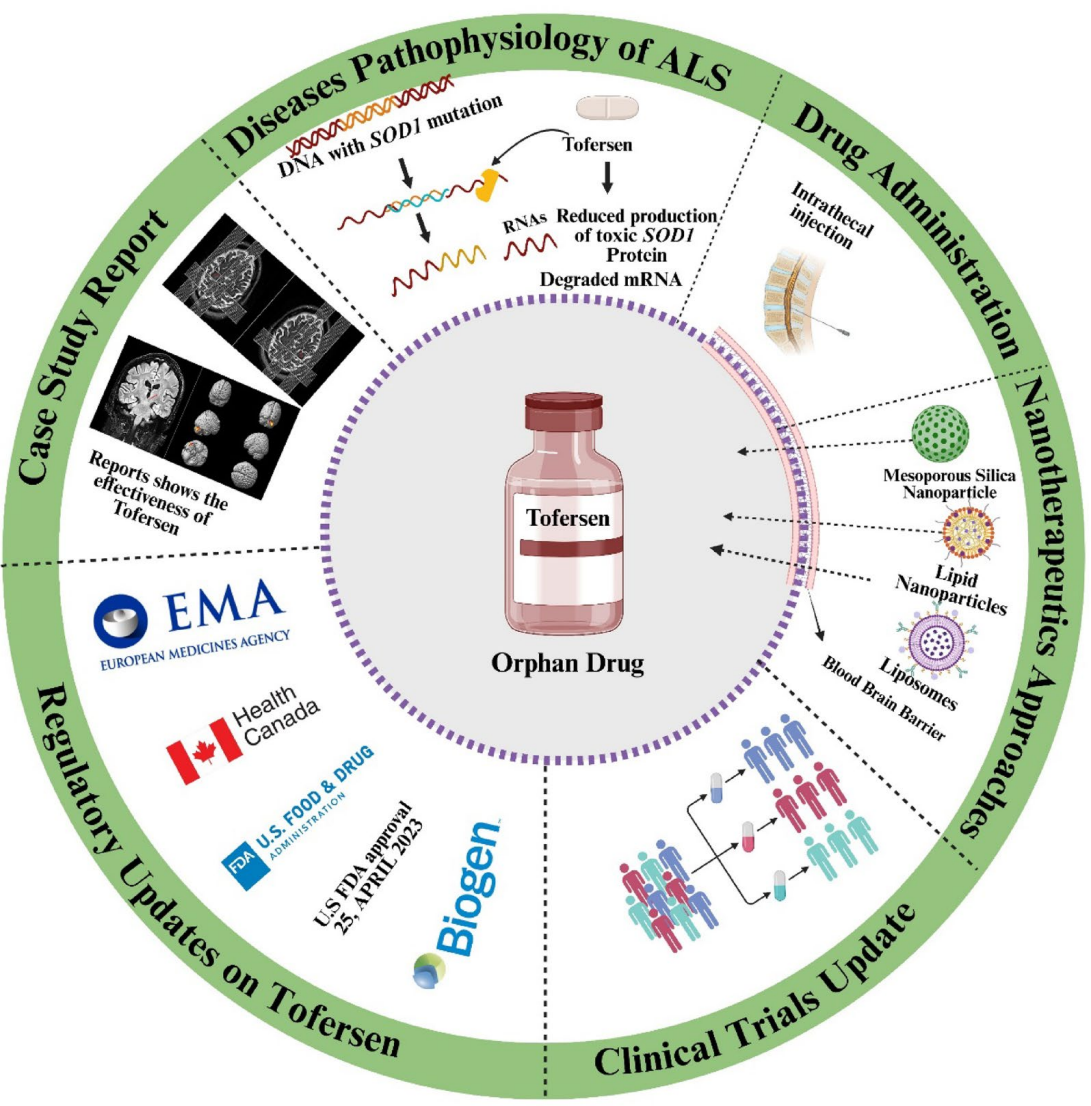

## Article Highlights

- This review provides a concurrent approach to nanotherapeutics and updates clinical trials for the therapeutic management of amyotrophic lateral sclerosis (ALS).
- It offers the latest regulatory updates on Tofersen, which is categorised as an orphan drug.
- The review also provides comprehensive information on the disease pathophysiology of ALS, single-cell molecular dynamics, case study reports, clinical trials, the physicochemical profile, and the regulatory status of Tofersen.
- Furthermore, it highlights major gaps in current nanotherapeutics approaches, offering valuable insights for future research.

## Introduction

Amyotrophic lateral sclerosis (ALS) is a progressive, fatal neurodegenerative disease primarily affecting motor neurons, leading to rapid motor impairment and loss of voluntary muscle control [1–4]. The global prevalence of ALS is estimated at 6.6 cases per 100,000 people, with recent U.S. studies suggesting a range of 7 to 9.1 cases per 100,000 [5–7]. The estimated number of individuals living with ALS in India is approximately 75,000 to 100,000 [8]. Despite extensive research efforts, there is currently no definitive cure for ALS [9]. However, various therapeutic interventions aim to manage symptoms and enhance patients’ quality of life. The primary symptom of this disease is degeneration of motor neurons, which compromise muscle control, speech, swallowing, and breathing functions [10, 11]. It manifests between 40 and 70 years of age; ALS symptoms encompass muscle weakness, twitching, and eventual paralysis. This disease has basically been characterized in two subtypes, such as sporadic amyotrophic lateral sclerosis (sALS) and familial amyotrophic lateral sclerosis (f-ALS), and differing inheritance patterns [12]. In this case 20 genes have been linked to f-ALS, with chromosome 9 open reading frame 72 (C9orf72) (40%), superoxide dismutase 1 (SOD1) (20%), focused ultrasound (FUS) (1–5%), and transactive response DNA binding protein (TARDBP) (1–5%) being the primary genes responsible for the majority of f-ALS cases [13]. While sALS constitute around 90% of cases, they occur randomly without a family history. Though a combination of genetic, environmental, and lifestyle factors are known to be implicated, the exact cause of sALS remains unclear. Both types exhibit similar symptoms, differing in familial history presence [14]. ALS is slightly more common in men than women, particularly at younger ages, but evens out with age [15].

To mitigate this disease, the United States Food and Drug Administration (US-FDA) has approved Tofersen (Qalsody), an antisense oligonucleotide for ALS therapeutics management under the orphan drug categories [16]. Recently, the regulatory agency (US-FDA) has approved a few more drugs, such as “Riluzole” and “Edaravone” for the treatment of ALS. For detailed information, refer to Table 1 [17, 18]. On the other hand, tofersen is an active ingredient in the proprietary name “Qalsody.” It is the first gene therapy approved for ALS, specifically for patients with the SOD1 genetic form of the disease, and represents a novel therapeutic intervention [19]. Tofersen only applies to a small subset of ALS patients (< 2%) with SOD1 mutations. Treatments starts with three doses given 2 weeks apart, followed by one dose every 4 weeks. Tofersen effectively reduces neurofilament-axonal injury and neurodegeneration in ALS, leading to positive outcomes [3].

**Table 1:** Comparative overview of FDA-approved drugs and investigational therapies for amyotrophic lateral sclerosis (ALS)

Drug/innovator	Mechanism of action/target genes	Route of administration/dosing frequency	Post marketing surveillance					Refs.
			Regulatory approvals & NCT No.	Adverse effect	Toxicity	Post-marketing Trials	Prescription status/brand name	
Riluzole/Rhone-Poulenc Rorer Pharmaceuticals	Inhibits glutamate release, blocks voltage-gated Na⁺ channels/GLT1, EAAT2 (glutamate transporters), Na⁺ channels, GluR	Oral/50 mg twice daily	12th Aug 1995	Nausea, dizziness, elevated liver enzymes	Elevated LFTs, rare hepatic failure Hepatotoxicity	Completed	Yes/Rilutek	[24,25]
Edaravone/Mitsubishi Tanabe Pharma Corporation	Free radical scavenger reduces oxidative stress/Indirectly affects SOD1, ROS, lipid peroxidation	IV infusion/14 days	05th May 2017	Gait disturbance, rash, headache	Potential nephrotoxicity	Completed	Yes/Radicava	[26,27]
AMX0035/Amylyx Pharmaceuticals	Inhibits ER stress and mitochondrial dysfunction/CHOP, BAX, BCL-2, ASK1 pathway, oxidative stress genes	Oral (powder suspension)/once daily	29th Sept 2022	Diarrhoea, nausea	–	PHOENIX Phase 3 ongoing	Withdrawn from U.S./Canada April 2024/Relyvrio (formerly AMX0035)	[28–30]
Tofersen/Ionis Pharmaceuticals (developed with Biogen)	Antisense oligonucleotide that binds SOD1 mRNA →degradation via RNase H/ SOD1 mutation	Intrathecal (lumbar puncture)/3 doses over 14 days, then every 4 weeks	25th Apr 2023	Lumbar pain, fatigue, myalgia	Neuroinflammation/No cardiotoxicity & nephrotoxicity reported	ATLAS trial (presymptomatic SOD1) ongoing	Yes/Qalsody	[19,31,32]
AP-101/AL-S Pharma AG	Monoclonal antibody against misfolded SOD1 facilitates clearance/ SOD1 (misfolded protein), possibly affecting the proteostasis network.	IV infusion Not finalised; in clinical trials/typically every 2–4 weeks	Not approved/NCT05039099	Infusion reactions, headache, fatigue (subject to trial confirmation)	Potential (ASO-related neuroinflammation	No post-marketing data	No/(monoclonal antibody in trials)	[33]
Jacifusen/Ionis Pharmaceuticals and Biogen	Custom-designed ASO targeting FUS mRNA, RNase H degradation/FUS gene mutation (ALS-FUS subtype)	Intrathecal injection via lumbar puncture escalation phase/bi-weekly (20 mg to 120 mg). Maintenance: usually 120 mg monthly	Not approved/NCT04768972	headache, post-lumbar-puncture headache, back pain, nausea	Suspected neurotoxicity	No post-marketing data	No/also known as ION363/Ulefnersen	[34,35]

Additionally, due to its landing in the market under orphan categories of drugs, there is a huge, marked demand, and no alternative medicine is available. However, some limitations have been recorded, such as procedural pain, post-lumbar syndrome, headache, myalgia, and arthralgia. This is discussed in the case study report and the pharmacovigilance study below. This review aims to explore the concurrent regulatory update on ALS to therapeutic management. Additionally, it highlighted the clinical trials and case study reports on ALS management associated with tofersen as a first-line drug.

## ALS background and unmet needs

ALS is a relentlessly progressive and fatal neurodegenerative disease marked by the selective loss of both upper and lower motor neurons, resulting in muscle weakness, paralysis, and ultimately respiratory failure [20]. The average survival following diagnosis ranges from 3 to 5 years, although this varies depending on the age of onset and disease progression rate. Despite extensive research efforts, the precise cause of ALS remains largely elusive, with evidence pointing to a complex interaction of genetic, environmental, and molecular factors. Notable mutations in genes such as SOD1, C9orf72, and TARDBP have been identified as contributors to both familial and sporadic ALS cases [21]. Currently, therapeutic options for ALS are limited, with FDA-approved medications like riluzole and edaravone offering only modest benefits by slowing disease progression or alleviating symptoms rather than reversing or preventing neuronal degeneration. This underscores a pressing unmet need for more effective, targeted treatments. The pathological heterogeneity of ALS further complicates the development of universal therapies.

**Fig. 1:**
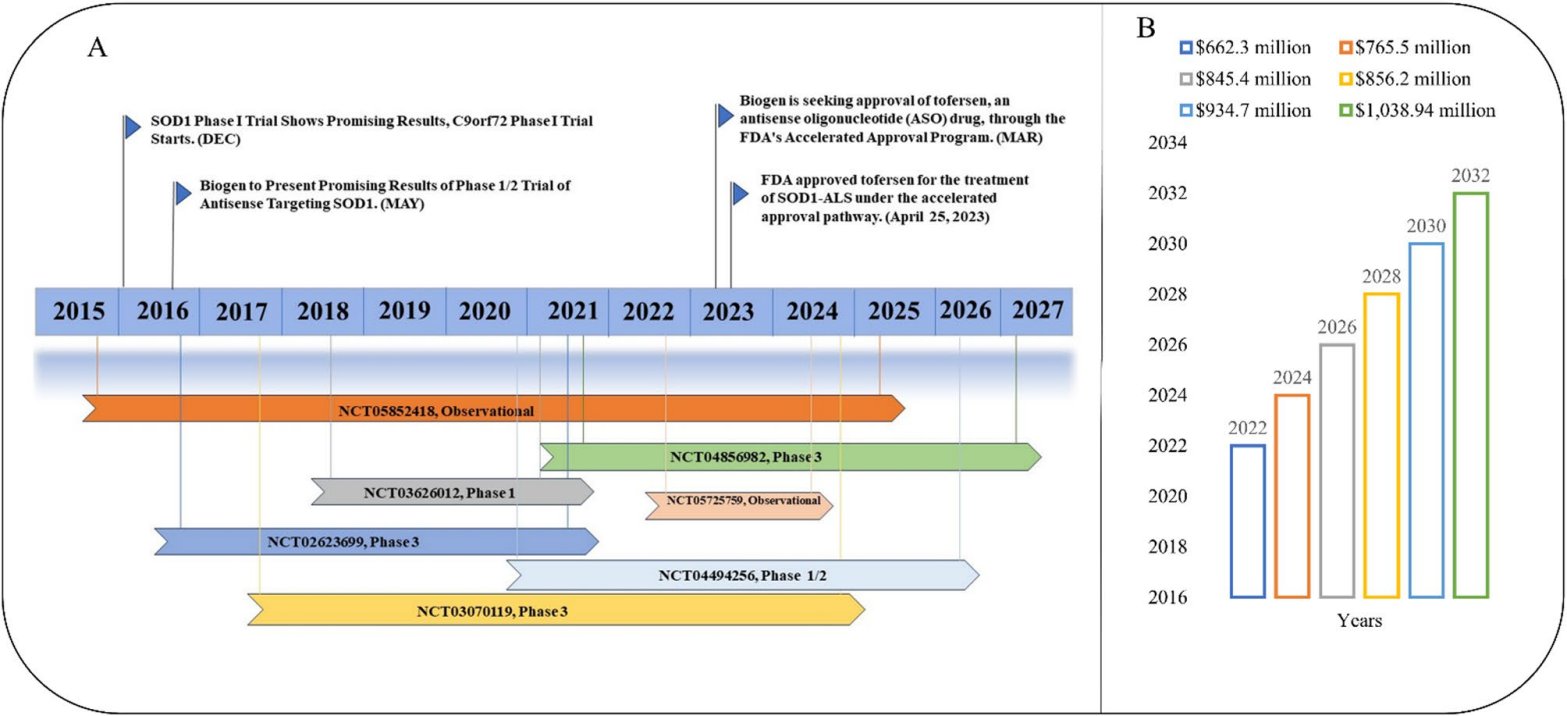
(**A**) The diagram illustrates the key milestones in the development of Tofersen- from its development as an Antisense oligonucleotide targeting SOD1 mRNA progression into clinical studies Phase I, II, III clinical trials evaluating its safety, tolerability, and efficacy to its accelerated approval [20]. (**B**) Coverage of the Global ALS market report. As of 2022, the pharmaceutical market for ALS was estimated to be worth $662.3 million. From 2023 to 2032, the market will increase at a CAGR of 4.6% and by 2032, it is anticipated that the market for ALS will be worth $1,038.94 million [20]

Consequently, there is an urgent demand for disease-modifying interventions that target fundamental molecular pathways, enhance patient quality of life, and prolong survival [22]. In years 2022, the pharmaceutical market for ALS was estimated to be worth $662.3 million. However, the predicted demand will increase by 4.6% in the upcoming year 2032, and the ALS market is anticipated to be worth $1038.94 million (see Figure 1) [23].

## Tofersen: mechanism and clinical data

### Molecular mechanisms

**Fig. 2:**
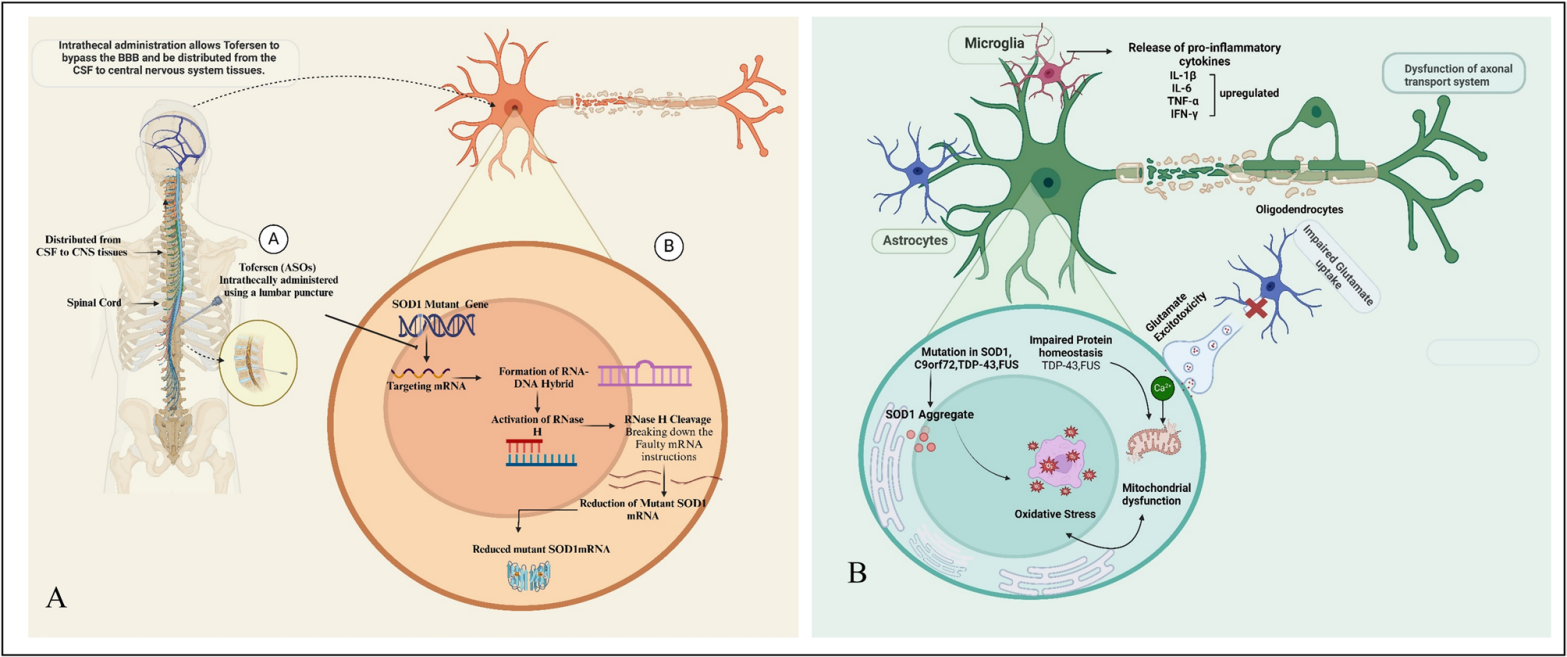
(**A**) This figure summarizes the multifaceted pathogenic mechanisms underlying ALS. Genetic mutations and environmental factors contribute to protein misfolding, glutamate excitotoxicity, oxidative stress, and impaired protein homeostasis. Neuroinflammation, glial activation, and axonal transport defects further exacerbate neuronal dysfunction and degeneration, highlighting the complexity of ALS pathology. (**B**) Tofersen is delivered via lumbar puncture directly into the cerebrospinal fluid (CSF), which is absorbed by the central nervous system (CNS). It selectively targets mutant SOD1 mRNA, creating an RNA-DNA hybrid complex that activates RNase H. This enzymatic process leads to the degradation of the targeted mRNA, thereby reducing the synthesis of pathogenic SOD1 protein and consequently slowing down the progression of the associated disease. (Figure generated from Bio Render, Trail Version)

Tofersen is a member of the antisense oligonucleotide (ASOs) class, which consists of short, synthetic single strands of DNA or RNA that change the regulation of mRNA by binding to a complementary sequence [36]. Tofersen effectively reduces the production of the SOD1 protein by directly binding to and mediating the breakdown of SOD1 mRNA generated from mutant SOD1 genes. Administered intrathecally into the Cerebrospinal fluid (CSF) surrounding the spinal cord, tofersen enters motor neurons and binds to SOD1 mRNA, forming an RNA-DNA hybrid [37]. This hybrid activates RNase H, leading to the cleavage of the RNA strand and subsequent degradation of mutant SOD1 mRNA (see Figure 2) [38, 39].

### Pathophysiology of diseases (ALS)

**Fig. 3:**
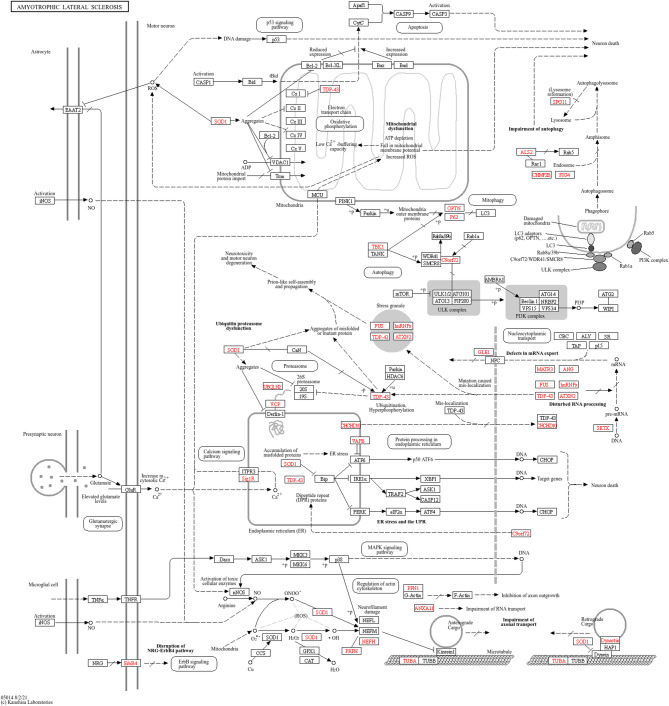
Pathophysiology of ALS. A complex interaction of molecular and genetic pathways causes ALS. Dysregulated glutamate excitotoxicity, induced by EAAT dysfunction, leads to neurodegeneration. More than 40 genes are responsible for the disease. Mutations in genes like c9orf72, TDP-43, FUS, and SOD-1 cause RNA metabolism abnormalities, oxidative stress, and microglial activation. (The image has been generated by the KEGG database, copy right premission granted ref No. Ref: 252675)

In the pathophysiology of ALS, various cellular and molecular mechanisms contribute to the degeneration of neurons, encompassing issues like mitochondrial dysfunction, impaired protein degradation, the formation of toxic protein aggregates, oxidative stress, and more [40, 41]. Over 40 genes have been linked to ALS, and mutations in four specific genes, namely C9ORF72, TARDBP [42], SOD1, and FUS, are responsible for over 70% of familial ALS cases (Table 2) [43]. Specifically, mutations in the SOD1 gene can produce mutant SOD1 protein, which may possess toxic properties, contributing to neuron degeneration [44]. Despite some mutant variants retaining partial or full dismutase activity, their presence is associated with ALS progression, indicating the involvement of other toxic characteristics (Figure 3). The disease’s progression typically begins in a specific area and then spreads throughout the motor system, which accounts for the disease’s unyielding progressive nature and suggests a possible ‘prion-like’ spreading mechanism. Although ALS is invariably fatal, there is significant variability in the age at which it begins, the pace of progression, the balance between upper and lower motor neuron involvement, and the extent of frontotemporal impact. Furthermore, ALS exhibits high genetic diversity, with over 20 genes currently associated with the condition [45].

**Table 2:** The table summarizes genes and pathways involved in ALS progression

Genes	Description	Pathways	Refs.
C9orf72	The most prevalent genetic etiology of f-ALS was found to be an aberrant extension of the GGGGCC hexanucleotide repeat in C9orf72	Protein homeostasis, RNA metabolism, Nucleocytoplasmic transport, Vesicle transport, Mitochondrial function	[46,47]
FUS	FUS protein aggregates are common in ALS patients	Protein homeostasis, RNA metabolism, DNA repair, Mitochondrial function	[48,49]
SOD1	SOD1 is a powerful antioxidant enzyme; At least 170 mutations in the SOD1 gene have been found to cause ALS	Protein homeostasis, Cytoskeleton dynamics, Mitochondrial function, Oxidative stress, Metal homeostasis	[50,51]
TDP-43	TDP-43 is the main component of cytoplasmic protein inclusions in ALS patients	Protein homeostasis, RNA metabolism, Stress granules	[52,53]
HnRNPA1/A2/B1	HnRNPs are relatively rare in ALS but are involved in the pathogenesis of ALS, perhaps through the combination with other common pathogenic genes (TDP-43)	RNA metabolism	[21,54]
C21ORF2	Over 75% of its mutations are linked to ALS risk.	DNA repair	[55,56]
CHCHD10	Mitochondrial dysfunction due to rare ALS-associated mutations	Stress granules	[57–59]
NEK1	Loss-of-function variants linked to f-ALS susceptibility	DNA damage response, mitochondrial function, cytoskeleton regulation	[60]
MATR3	S85C mutation in MATR3 is a genetic contributor to ALS	RNA metabolism, RNA binding and processing dysfunction	[61,62]
TAF15	Its accumulation is associated with neurodegeneration in ALS	RNA metabolism, abnormal stress granule formation	[63–65]
EWSR1	Overexpression of wild-type EWSR1 leads to neurodegeneration	RNA processing, gene expression regulation	[66]
ATXN2	The expansion of ATXN2 intermediate length polyglutamine increases the risk of ALS	RNA metabolism, stress granule assembly, toxic polyglutamine repeat expansion	[67]
TIA1/TIAR	TIAR may be involved in neuronal cell death after ischemia, while an increased risk for TIA1 LCD mutations was found in ALS patients	Stress granule formation, RNA metabolism, phase separation	[68,69]
TBK1	A mutation in TBK1 is the main genetic cause of ALS/FTD comorbidities (10.8%), while it is less associated with ALS alone (0.5%)	Autophagy, inflammation, protein degradation pathways	[70,71]
TUBA4A	Mutations in TUBA4A are associated with f-ALS, and all patients with TUBA4A mutations experience spinal seizures accompanied by upper and lower motor neuron signs	Cytoskeletal instability, axonal transport	[72]
CCNF	Increases TDP-43 aggregation; rare ALS cause	A ubiquitin-proteasome system, proteostasis	[73]
KIF5A	Cargo-binding mutations cause ALS	Axonal transport, microtubule motor protein dysfunction	[74]
ANXA11	Gain-of-function mutations lead to protein aggregation	Vesicle trafficking, stress granule dynamics	[75]
GLT8D1	Glycosyltransferase gene associated with f-ALS	Golgi function, vesicle trafficking abnormalities	[76]
SPG11	It is because of juvenile ALS and spastic paraplegia	Lysosomal/autophagic pathways, axonal maintenance	[77]
miRNAs	miR-27a, -34a, -124, -142-5p, -155, -338-3p are ALS biomarkers	Post-transcriptional gene regulation, modulation of neuroinflammation and apoptosis	[78–82]
lncRNAs	NEAT1_2 can regulate the function of ALS-associated RNA-binding proteins in the early stage of ALS	Regulation of gene expression, chromatin remodelling, acting as molecular scaffolds or decoys	[83–85]
UA	Higher ALS mortality is associated with low levels	Antioxidant, a potential protective factor against oxidative stress	[86]
CL	Alterations in CL levels may also reflect the loss of mitochondrial integrity observed in several ALS models	Mitochondrial membrane stability, apoptosis, energy metabolism	[22]
CHIT1	An elevated level of CHIT1 in the cerebrospinal fluid of ALS patients has been indicated	Marker of neuroinflammation, microglial activation	[87]
NfL	Serum NFL is positively correlated with disease progression, while a higher NFL level indicates a shorter survival period	Biomarker for axonal damage and disease progression in ALS	[88]

### Clinical trials updates

The VALOR clinical trial, registered as NCT02623699, investigated the efficacy of tofersen, an ASO, in treating SOD1 ALS [89]. From March 2019 to July 2021, the study enrolled 72 adults with SOD1 ALS who were randomly assigned to receive either tofersen or a placebo intrathecally for 24 weeks. The primary goal was to assess changes in ALS Functional Rating Scale-Revised (ALSFRS-R) score by week 28 in participants with faster disease progression. Secondary objectives included analyzing alterations in SOD1 protein concentration, neurofilament light chain concentration, slow vital capacity, and handheld dynamometry. While no significant clinical improvement was observed with tofersen over placebo at 28 weeks, a 52-week analysis suggested potential benefits associated with early initiation of tofersen compared to delayed initiation [3]. Statistical analysis used a joint rank test to compare treatment effects, considering functional decline and survival. An open-label extension study (NCT03070119) was conducted to evaluate the effects of early versus delayed tofersen initiation [90]. Participants who started tofersen in VALOR comprised the “early-start cohort,” while those transitioning from placebo in the extension formed the “delayed-start cohort.” Various outcomes, including ALSFRS-R score, SOD1 protein concentration, neurofilament light chain concentration, slow vital capacity, and safety measures, were assessed in this extension study [91]. Combining data from VALOR and its extension sheds light on sustained reductions in SOD1 protein concentration and neurofilament light chain concentration with tofersen over time. Moreover, differences in clinical outcomes between the early-start and delayed-start cohorts suggested potential advantages of early treatment initiation [46]. Overall, the findings from this trial offer valuable insights into tofersen’s impact on SOD1 ALS progression and advocate for further investigation in this field. For more details on clinical trials, refer to Table 3.

**Table 3:** Concurrent clinical trials updated on recently approved drug (Tofersen) for the therapeutic management of ALS

NCT number	Objectives	Drug	Primary outcome	Routes of administration/ doses	Study type and phase	Country origin	Collaborators	Sponsor	No. of patients
NCT03070119	To assess the long-term safety and tolerability of tofersen in participants diagnosed with ALS and confirm the SOD1 mutation	Tofersen	The primary outcome has been designed to track the number of participants for up to 364 weeks by experiencing AEs and serious adverse events, if any	Intrathecal injection Doses:100 mg	Interventional/ Phase 3	United States of America	Ionis Pharmaceuticals	Biogen	139
NCT02623699	The study aims to evaluate the safety, tolerability, and pharmacokinetics of escalating doses of tofersen in adults with ALS and confirmed SOD1 mutations	Tofersen	Parts A and B will monitor adverse events, lab abnormalities, vital signs, and neurological examinations alongside the pharmacokinetic parameters of BIIB067	Intrathecal injection Doses: 20, 40, 60 or 100 mg	Completed/Phase 3	United States of America	Ionis Pharmaceuticals	Biogen	176
NCT04856982	This study aims to assess the effectiveness of presymptomatic adults carrying SOD1 mutation with elevated neurofilament (NF) levels	Tofersen	Parts B and C will measure the percentage of participants developing clinically manifest ALS within 24 months and 1 year, respectively, from the baseline of Part B	Intrathecal injection Doses:100 mg	Recruiting/Phase 3	United States of America	NA	Biogen	150
NCT04972487	The goal of this early access program (EAP) is to offer tofersen to eligible individuals with ALS linked to a SOD1 gene mutation before an alternative access method is available, aiming to meet the urgent medical needs of this group	Tofersen	Underway	Intrathecal injection	Approved for marketing/NA	United States of America	NA	Biogen	NA
NCT03626012	This study aims to assess the safety and tolerability of BIIB078 in adults with C9ORF72-ALS	BIIB078	The study will track AEs and SAEs from baseline through the end of the study, approximately Day 323	Intrathecal injection Doses: 20, 40, 60 or 100 mg	Completed/Phase 1	United States of America	NA	Biogen	106
NCT05725759	This study assesses personalised rehabilitation’s effects on SOD1 ALS patients in the tofersen access program, utilizing outpatient therapy and individualized programs based on initial assessments	Rehabilitation	The study will measure changes in ALSFRS-R scores from baseline to 12 months, assessing functional severity across bulbar function, gross and fine motor skills, and respiratory function	NA	Enrolling by invitation/NA	United States of America	NA	Washington University School of Medicine	10

### Adverse effects (ADRs)

The adverse effects associated with tofersen were deeply investigated. Among the participants, 96% experienced adverse events, with about 18% being serious and 1% fatal [3, 92]. Notably, 6% discontinued the treatment due to its adverse events. Common adverse events included headaches (46%). Procedural pain (57%), falls (24%), and back pain (21%). Serious events like respiratory failure (1%), pneumonia aspiration (3%), and pulmonary embolism (4%) underscored the need for careful monitoring [92].

## Case study report (pharmacovigilance)

### Case study: -1

A 61-year-old man had been experiencing growing distal weakness in his lower limbs for the past two years. His social and medical histories were not instructive. He mentioned a family history of ALS, stating that his brother passed away at the age of 59 from the disease, which is characterized by progressive lower limb paralysis and respiratory impairment that necessitates noninvasive ventilation (NIV) five years after onset. The patient’s neurological examination showed hypotrophy and mild weakness in both lower limbs, although upper limb and bulbar function were initially unaffected. All four limbs were affected by widespread fasciculations. All limbs had quick, deep tendon responses, with the left side having the strongest and no stiffness. The diagnosis of ALS was made using the updated El Escorial Criteria following a comprehensive diagnostic work-up that included neurophysiological investigations that showed active denervation and reinnervation potentials in three body locations. Fascinatingly, MRI showed UMN involvement as a decrease in the pyramidal bundle’s fractional anisotropy (FA) values in tractography acquisitions and hypo intensity in the bilateral motor cortex on T2-weighted images. Transcranial MEPs revealed reduced amplitudes with normal latencies and central motor conduction times in the lower limbs [93].

Cognitive profiles were found to be normal after extensive neuropsychological testing. The first impairment of respiratory function was asymptomatic [forced vital capacity (FVC): 70%].

**Fig. 4:**
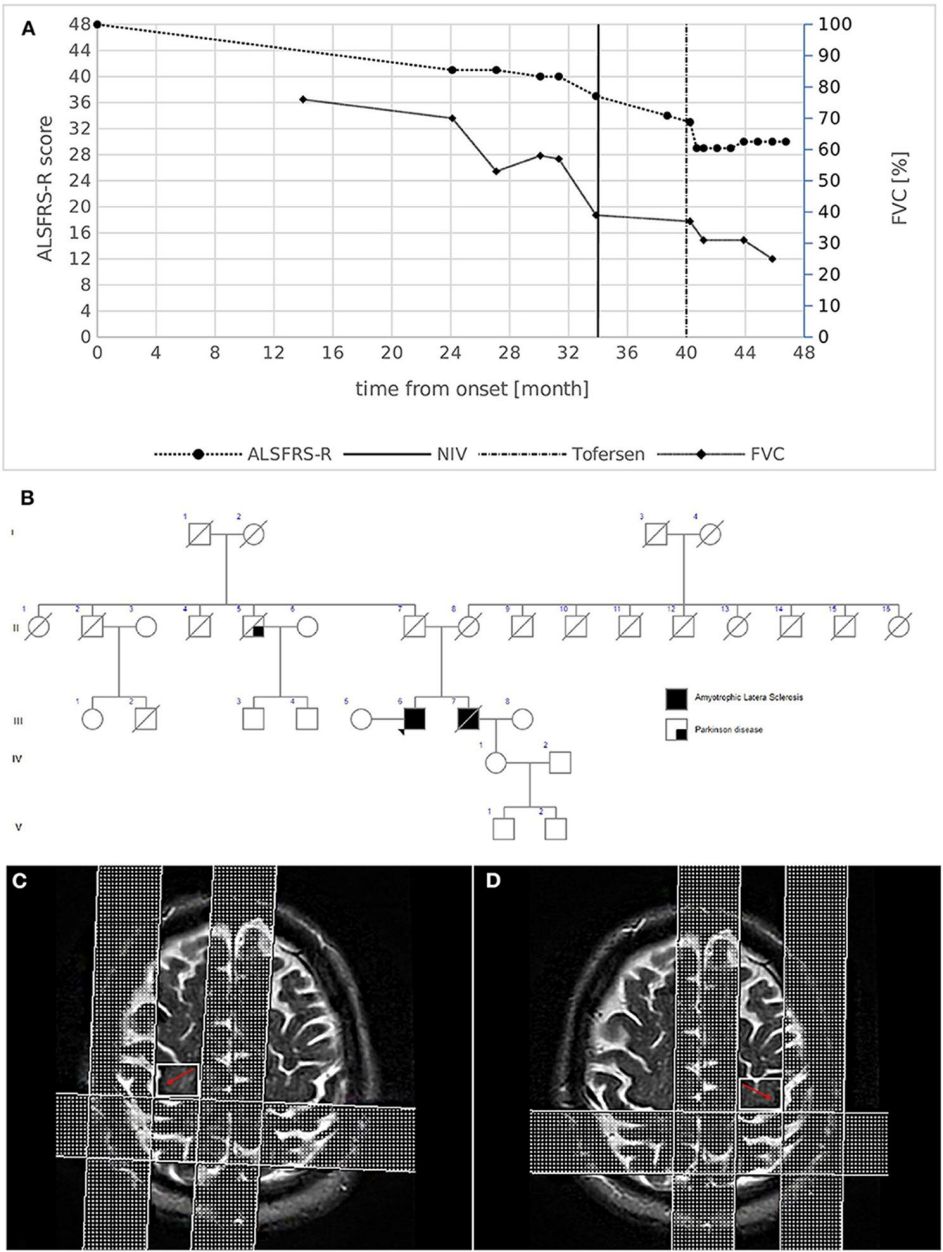
(**A**) Disease course of case 1. The graph shows ALSFRS-R total score (left) and FVC % (right) decline from disease onset. The bold vertical line represents the time when NIV was initiated, while the dashed vertical line represents the beginning of intrathecal Tofersen administration. (**B**) Family pedigree of case 1. Filled circle/square, affected individuals; open circle/square, unaffected individuals; arrow, proband; diagonal line, deceased individuals. The proband's mother died at 79 years of age from HCV infection complications and chronic COPD; the father died at 94 years of age after a hip fracture. A paternal uncle died at an advanced age because of Parkinson's disease. (**C**, **D**) Brain MRI images of case 1. MRI T2-weighted images revealing the hypointensity along the bilateral motor cortex (red arrows) [93].

Further, a progression rate of 0.29 points each month, the updated ALS functional rating scale (ALSFRS-R) score was 41/48. NIV was started to assist respiratory function after the weakness had progressed to the upper limbs a year later (Fig. 4A-D). Forty-eight months following the onset of symptoms, the patient is still alive. He is currently receiving treatment with intrathecal Tofersen (early access program) for respiratory impairment (FVC 25%) without bulbar involvement, weakness in four limbs with major LMN symptoms, and other conditions [93].

### Case study: -2

**Fig. 5:**
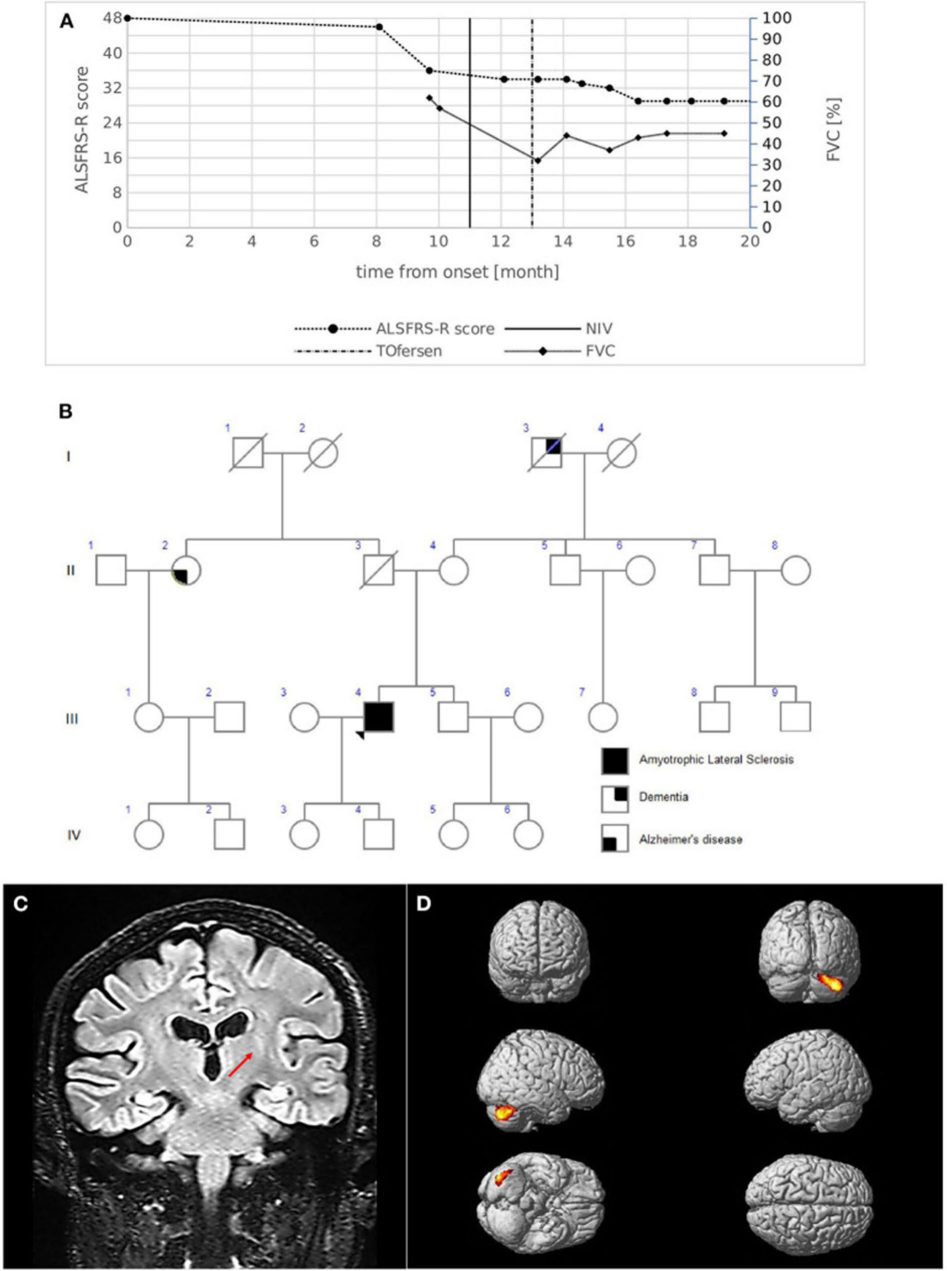
(**A**) Disease course of case 2. The graph shows ALSFRS-R total score (left) and FVC % (right) decline from the onset of symptoms. The bold vertical line represents the time when NIV was initiated, while the dashed vertical line represents the beginning of intrathecal Tofersen administration. (**B**) Family pedigree of case 2. Filled circle/square, affected individuals; open circle/square, unaffected individuals; arrow, proband; diagonal line, deceased individuals. The proband's father died at 56 years of age of colon cancer; the mother is still alive; a 51-year-old brother is alive and in good health and is currently undergoing psychological counseling to evaluate genetic testing. Maternal grandfather had dementia which started at an advanced age; a paternal aunt had Alzheimer's disease with onset at 75 years. (**C**) MRI coronal acquisition showing FLAIR hyperintensity of left cortico-spinal bundle; the red arrow indicates the peculiar involvement of the internal capsule segment. (**D**) ^18^FDG-PET images Glass brain rendering of the comparison between subject 2 and 40 healthy controls (HC). The cluster showing a statistically significant relative hypermetabolism in subject 2 compared to HC is projected on the brain surface (height threshold *p* < 0.001; *p* < 0.05 FWE-corrected at cluster level) [93].

A 53-year-old man arrived with proximal weakness in his upper limbs and walking issues that had been persistent for 8 months. He had a medical history of diverticular disease, bipolar disorder, asthma, and essential tremor (ET). A neurological examination revealed hypotrophy of the intrinsic hand muscles and the right calf, along with slight proximal weakness in the upper limbs and modest weakness in the lower limbs, primarily on the right side. The bulbar function remained entirely unchanged. All limbs had rapid, deep tendon reflexes and bilateral Hoffman and Babinski signals. There was no spasticity. There was no documented family history of ALS (Fig. 5B). Based on the updated El Escorial Criteria, ALS was diagnosed after diagnostic testing. According to electrophysiological investigations, three body regions exhibited active denervation and reinnervation potentials. According to transcranial MEPs, the right upper limb had a longer central motor conduction time with a higher latency and a smaller amplitude. Bilateral motor cortex hypo intensity and left cortico-spinal bundle FLAIR hyperintensity were observed on MRI T2-weighted images (Fig. 5C). Reduced FA values were found in the cortico-spinal tract acquisitions by MRI tractography. Both cerebellar hemispheres had regions of hypermetabolism, with the right lobe showing the highest prevalence, according to 18 FDG-PET (Fig. 5D) [94].

### Case study: -3

A case study examined a female patient with SMA type 3 who was 61 years old. Since 20th Apr 2018, she has had 17 injections of nusinersen in 54 months after being diagnosed with SMA at the age of 15. Following the suggested dosing schedules, nusinersen was given four loading doses on treatment days 0, 14, 28, and 63, followed by major maintenance doses every four months. After retrieving CSF, 12 mg of nusinersen was administered intrathecally via lumbar puncture in each administration. No clinically relevant side effects have yet been noted during treatment. The patient had no previous experience of spinal surgery and no severe scoliosis. CSF cytology results prior to nusinersen treatment revealed macrophages with a typical structure. The patient’s CSF showed no intrathecal production of immunoglobulins (Igs), a normal leucocyte cell count, and normal total protein and albumin contents. On treatment day 63, macrophages exhibited unclear inclusions during the loading phase. These inclusions were then found in treatment months 14, 26, and 30, and they have been there continuously since treatment month 38. No discernible changes were seen throughout treatment in the leucocyte cell count, total protein and albumin concentration, or the presence of Igs in CSF (Table 4) [93, 94].

**Table 4:** Cerebrospinal fluid findings suggested treatment initiation with Nusinersen and Tofersen

Biochemical Parameter	Reference values	Patient 1—SMA:			Patient 2—SOD1-ALS:		
		T1	T2	T3	T1	T2	T3
Leucocyte count (MPt/L)	< 5	3	4	1	1	3	**21**
Protein (mg/L)	150–450	249	262	214	**483**	**764**	**1235**
Albumin (mg/L)	< 350	129	137	134	268	**558**	**879**
Albumin ratio (× 10^− 3^)	(4+ age/15)	3.37	3.58	3.41	5.6	**12.85**	**18.65**
IgG (mg/L)	10–30	14.1	12.8	12.53	**44.48**	**69.09**	**115.63**
IgG synthesis (%)	Negative	0	0	0	1.5	0	0
IgA (mg/L)	1–3	1.29	1.58	1.74	**5.81**	**10.78**	**17.94**
IgA synthesis (%)	Negative	0	0	0	0	0	0
IgM (mg/L)	0.5–1.5	< 0.5	< 0.5	< 0.12	0.33	**1.82**	**12.26**
IgM synthesis (%)	Negative	0	0	0	0	0	62.6
OCBs (CSF/Serum)	Negative	Type 4	Type 4	Type 1	Type 4	Type 1	Type 4
Lymphocytes (%)	50–90	**93**	88	**97**	**94**	85	85
Monocytes (%)	10–50	**6**	**9**	**1**	**3**	**9**	10
Macrophages with unspecified inclusions	None	−	+	+	−	+	+

T1: treatment-naive; T2: month 6 of nusinersen treatment/month 2 of tofersen treatment (after completed loading phases); T3: month 54 of nusinersen treatment/month 9 of tofersen treatment for SOD1-ALS., CSF samples were also collected at three stages: T1 indicates the treatment-naive state before starting tofersen, T2 marks the CSF status at month 2 of tofersen treatment after the loading phase was completed, and T3 shows the CSF profile at month 9 of tofersen treatment, representing the ongoing maintenance phase. The clinical data credited to Vidovic et al. [94] under license; visit http://creativecommons.org/licenses/by/4.0/ [94]

### Case study: -4

This patient, a 39-year-old woman, was diagnosed with SOD1-ALS (c.358-10T>G) in 2022 in February and was given 11 injections of tofersen in 9 months since 22nd Oct 2022. The patient received tofersen in accordance with the recommended dosing schedules, with three loading doses on treatment days 0, 14, and 28 and maintenance doses each month. Each injection contains 100 mg of tofersen, which is administered intrathecally via lumbar puncture after CSF is obtained. No further clinically significant adverse events have happened, with the exception of a pseudo-radicular pain incident following the initial injection. CSF cytology results prior to tofersen administration showed normal albumin content, leucocyte cell count, and macrophages without inclusions. There was just a small rise in CSF total protein and IgG content. On day twenty-eight, after the start of therapy, macrophage inclusions were initially seen. Further, mild pleocytosis was observed, and since treatment month three, there had been an increase in the amounts of albumin and total protein. At the same time, intrathecal production of the immunoglobulin M antibody (IgM) was seen starting in treatment month 5 and gradually increased by treatment month 9. Prior to the loading phase and during treatment month nine, there were equal numbers of matched oligoclonal bands (OCBs) in CSF and serum (Type 4) [93, 94].

## Tofersen: pharmacokinetics and pharmacodynamics profile

### Pharmacokinetics

The pharmacokinetic study of tofersen investigated its absorption, distribution, metabolism, and elimination properties. When administered intrathecally into the CSF, tofersen, also known as QALSODY, facilitates distribution throughout CNS tissues. The highest concentration in the CSF, termed maximum CSF trough concentration, occurs after the third dose, marking the conclusion of the loading phase. Subsequent monthly dosing does not lead to significant accumulation in the CSF [3]. Moreover, the drug transitions from the CSF into the systemic circulation, with a median time to maximum plasma concentration (Tmax) ranging from 2 to 6 h [39]. Notably, there is no noteworthy accumulation of tofersen in plasma following monthly maintenance dosing. The case of postmortem examination of tissue from treated patients indicates the distribution of intrathecally administered tofersen within CNS tissues [37]. However, the elimination of tofersen is primarily metabolized via exonuclease-mediated hydrolysis and, subsequently, does not impact cytochrome P450 (CYP450) enzyme activity [95]. The median plasma volume of distribution was estimated at 50.9 L (119% CV) in studies 101 and 102 and 40.67 L (130% CV) in the 100 mg dose group. PK analysis demonstrates that intrathecally administered tofersen is widely distributed into CNS tissues and is rapidly transferred from CSF to systemic circulation [96]. This is followed by tofersen, which is bound to human plasma proteins (≥ 98% bound) at clinically relevant or higher plasma concentrations (0.1 and 3 g/ml), which limits glomerular filtration and reduces urinary excretion of the active substance. The elimination half-life was measured in the CNS tissue of cynomolgus monkeys and found to be 31 to 40 days. The median plasma clearance was estimated at 8.32 L/h. (60.6% CV) in studies 101 and 102; it was 5.73 L/hhr.0% CV) at 100 mg dose [97].

### Pharmacodynamics

In Study 1 Part C, involving patients with SOD1-ALS, the impact of tofersen on total CSF SOD1 protein, serving as an indirect measure of target engagement, was assessed. By Week 28, the towers-treated group exhibited a notable reduction of 35% (geometric mean ratio to baseline) in total CSF SOD1 protein compared to a mere 2% decrease in the corresponding placebo recipients within the ITT population [3]. This difference, reflected in a 34% higher geometric mean ratio for tofersen compared to placebo, was statistically significant (nominal *p* < 0.0001). Additionally, the effect of tofersen on neurofilament proteins, particularly plasma NfL, a blood-based biomarker indicative of axonal injury and neurodegeneration, was examined. By Week 28, QALSODY-treated subjects experienced a substantial 55% reduction (geometric mean ratio to baseline) in mean plasma NfL, contrasting with a 12% increase observed in the placebo group within the ITT population. The difference in geometric mean ratios between QALSODY and placebo was significant (60%; nominal *p* < 0.0001) [98]. Notably, the decline in plasma NfL persisted until approximately day 113 and was accompanied by similar reductions in phosphorylated neurofilament heavy chain (pNfH) and CSF compared to plasma. In the case of cardiac electrophysiology, it was observed that at the maximum approved recommended dosing regimen, QALSODY did not lead to any clinically relevant prolongation of the QTc interval [97].

## Limitations

### Drug delivery

The intrathecal administration of tofersen necessitates lumbar puncture for direct distribution into the cerebrospinal fluid, which presents inherent clinical and logistical difficulties. The complexity and burden of treatment delivery are increased by the requirement for specialized clinical infrastructure and skilled people for intrathecal administration [99]. This delivery method limits the therapy to specialized academic or clinical centers prepared for such procedures, potentially restricting patient access. The treatment protocol involving multiple lumbar punctures, initial loading doses followed by maintenance doses, further intensifies patient visits and procedural risks. Patients may experience adverse events related to the delivery method, including serious neurological adverse events such as myelitis, radiculitis, aseptic meningitis, and intracranial hypertension, with approximately 7% of treated patients experiencing serious neurologic events, some necessitating treatment discontinuation [100]. Moreover, despite reductions in biomarkers such as CSF SOD1 protein and plasma neurofilament light chain, tofersen has not demonstrated statistically significant clinical improvements in key functional measures in initial trials, which raises questions about clinical efficacy relative to delivery risks and complexities [101].

### Clinical

Tofersen is effective in reducing neurodegeneration biomarkers like NfL and NfH in CSF and serum. However, various clinical limitations are associated with treatment. The most significant limitation may be the fact that biomarker improvements have little association with clinical outcomes [102]. Levels of neurofilament light chain (NfL) and neurofilament heavy chain (NfH) continued to reduce, while ALSFRS-R scores dropped further, indicating that disease progression has not been halted [103]. No statistically significant correlation was found between changes in biomarkers and functional improvement, suggesting a potential disconnect between biochemical response and clinical benefit [104] A clinical study reported by Newman et al. (2025, reduction in CSF SOD 1 protein and plasma neurofilament light chain (NfL) consistently preceded measurable clinical benefit in the VALOR and Open-label extension studies. Additionally, during clinical phase III, double-blind, placebo-controlled VALOR (Clinical Trials. gov identifier: NCT02623699), the neurofilament levels have also been assessed as exploratory outcomes, with significant reductions observed in cerebrospinal fluid (CSF) on day 92 in the group treated with 100 mg of Tofersen compared with no change in the placebo group [105].

Additionally, neuroinflammatory markers such as Chitinase-3-like protein 1 (CHI3L1) and SerpinA1 increased progressively during treatment, leading to questions about the ASO-induced neuroinflammation. This inflammatory response could either reflect disease progression or be a result of repeated intrathecal delivery and ASO exposure. Serious side effects were present, including aseptic meningitis in 11% of patients, raising safety concerns associated with longer courses of treatment [106]. Further, surrogate endpoints have played a significant role in accelerating drug development and regulatory approval processes for serious diseases like ALS. The FDA’s accelerated approval pathway allows drugs to be approved based on surrogate markers “reasonably likely” to predict clinical benefit, thereby enabling earlier patient access to promising therapies [107]. However, this approach has sparked debate within the ALS community and broader regulatory landscape. Critics argue that approving treatments without a clear demonstration of clinical efficacy risks exposing patients to potential adverse events and financial burdens without proven benefit, while advocates emphasize the urgent need for new therapies in a fatal disease with limited options [108]. The case of tofersen exemplifies this tension—as it has been granted accelerated approval based on biomarker and early-phase data despite equivocal clinical trial results to date [109]. The findings from the VALOR and OLE studies have prompted the ALS research community to reconsider traditional clinical trial designs. Specifically, the standard six-month study duration may be insufficient to capture the full therapeutic benefit of investigational treatments in ALS. Extending trial length could be essential to more accurately evaluate treatment efficacy [110]. Furthermore, these results highlight the potential role of neurofilament biomarkers as surrogate endpoints in future placebo-controlled trials, given the observed delay between the initiation of therapeutic effect and the manifestation of measurable clinical benefit [111].

## Beyond tofersen: future therapeutic platforms

Tofersen sets a precedent as the first ASO therapy to receive accelerated approval for SOD1-mutated ALS, but the dramatic development of new therapies proceeds rapidly [112]. More ASOs, including BIIB078 and Wave Life Sciences’ WVE-004, specifically target C9orf72 repeat expansions, an area believed to be the most common genetic cause of ALS and frontotemporal dementia [113]. Initial trials have been conducted, such as NCT03626012 (BIIB078) and NCT04931862 (WVE-004); however, while they investigate the possibility of using these compounds to decrease toxicities by reducing RNA foci and dipeptide repeat proteins, as indicated, BIIB078 was withdrawn because of inefficacy [114, 115]. In parallel, one can expect CRISPR/Cas9 and base-editing development platforms for durable specificity in mutation corrections for genes associated with ALS. Examples include exploration of in vivo CRISPR-based approaches by Intellia Therapeutics and Editas Medicine for neurodegenerative disorders, while Noviome Bio and AskBio have begun preclinical programs for ALS [116]. There is a notable preclinical effort in CRISPR-mediated SOD1 knockout studies, associated with prolonged survival of SOD1-ALS mouse models. Progressive fast-moving programs are all heading toward the first-in-human studies, irrespective of the fact that no CRISPR-based therapies for ALS have yet advanced to clinical trials [117]. Both these emerging platforms, whether ASO or genome editing, take the therapeutic management of diseases out of symptomatic treatment into precision, disease-modifying therapeutic, and possible curative development strategies. This is critical to bolster the therapeutic scope beyond Tofersen to encompass the full momentum of innovation and the diverse genetic landscape of ALS.

### Therapeutic strategies in ALS

With a lumbar puncture, tofersen is given intrathecally. To deliver medication to the area surrounding the spinal cord, a lumbar puncture entails inserting a long, thin needle into the lower back’s skin and underlying structures [99]. ASOs need to be injected directly into the CNS since they cannot easily cross the blood-brain barrier (BBB). Ten milliliters of the patient’s CSF should be removed before giving them a dosage of tofersen. This process lowers the possibility of elevated intracranial pressure and avoids fluid overload. It is advised that patients get tofersen by gently injecting the drug for one to three minutes using the same lumbar needle that was used to remove the CSF [118].

### Nanotherapeutic strategies in ALS

Wang et al. [119], highlighted the recent advances in nanotechnology, offering a promising solution for ALS by enabling targeted drug delivery to the CNS despite the BBB obstacle [119, 120]. Generally, small drug molecules and lipophilic drugs have a molecular weight between 200 and 600 Da, and a particle size below 200 nm is acceptable to cross the BBB [121]. A range of nanoparticle variants, including liposomes, polymeric nanoparticles, and inorganic nanoparticles, have been investigated for ALS treatment, each offering unique drug delivery advantages [122]. Liposomes effectively cross the BBB, delivering drugs to the CNS. Polymeric nanoparticles provide controlled drug release, and inorganic nanoparticles like gold have properties useful for imaging and drug delivery [123, 124]. Despite promising preclinical results, addressing safety, biocompatibility, delivery efficiency, and production costs is crucial before the widespread clinical use of nanoparticles in ALS treatment. Yet, nanotechnology offers significant potential as a therapeutic approach, bringing hope for overcoming BBB drug delivery challenges [125, 126]. Recently, researchers have explored nanotherapeutic drug delivery systems for effectively ameliorating ALS. However, the traditional therapeutic medication is limited for ALS patients due to it being available in orphan drug categories [127]. Apart from that, a few other categories of drugs, such as pioglitazone, have shown promising therapeutic potential for ameliorating LAS. The nanocarrier drug delivery system showed a promising approach for the therapeutic management of LAS and easily crosses the BBB [128].

### Mesoporous silica nanoparticle

**Fig. 6:**
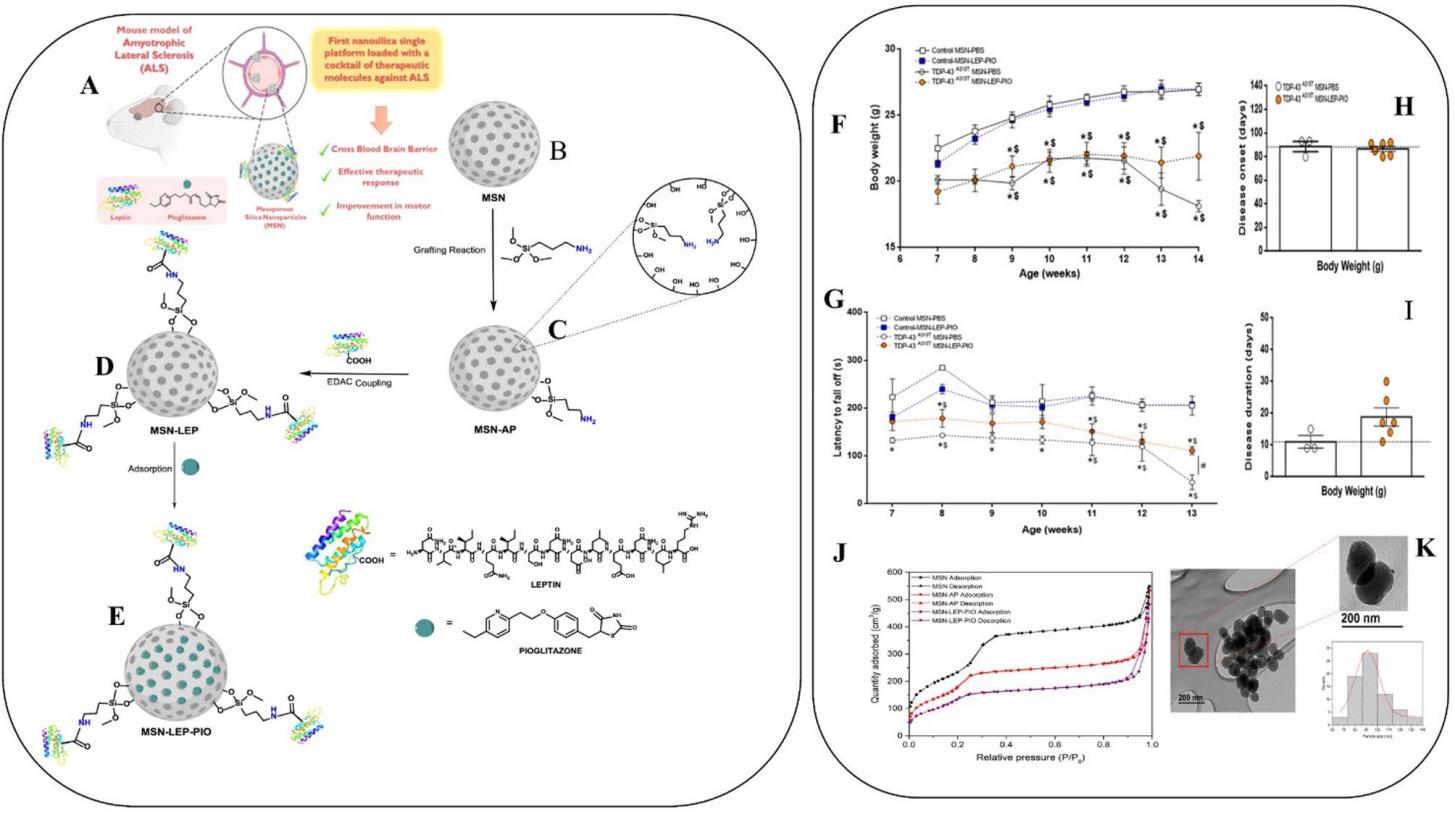
( **A**) Schematic illustration depicts mesoporous silica nanoparticles (MSNs) functionalization for ALS therapeutic applications in a mouse model. Initially, MSN silica nanoparticles are grafted with MSN-AP (**B**, **C**), followed by EDC coupling to conjugate MSN-LEP with pioglitazone and leptin (**D**, **E**) therapeutic agents. Treatment with MSN-LEP-PIO, initiated at the asymptomatic stage of the disease, significantly improves motor function in TDP-43 transgenic mice. (**F**) Beginning at week 7, the body weight of TDP-43 A315T mice and wild-type (WT) controls receiving intraperitoneal (IP) treatment with either MSN-LEP-PIO or PBS was monitored over time. There were no appreciable variations in the starting body weight or length of illness between TDP-43 A315T mice treated with MSN-LEP-PIO and PBS. (**G**) Longitudinal behavioral evaluations of motor performance in TDP-43 A315T mice getting MSN-LEP-PIO or PBS and WT controls showed that the MSN-LEP-PIO-treated groups significantly improved. The data is shown as mean ± SEM. Two-way ANOVA statistical analysis revealed significant variations (*p < 0.05) between the MSN-LEP-PIO along with PBS groups in TDP-43 A315T mice and WT controls. Several treatment groups are depicted in the graphs: TDP-43 A315T-MSN-LEP-PIO (*n* = 6, orange circles, dashed line), control-MSN-LEP-PIO and control-PBS (*n* = 3, white squares, solid line). In mice given MSN-LEP-PIO or PBS, the average illness start was computed based on changes in body weight. (**H**) In WT and TDP-43 A315T mice, disease duration was defined as the interval from the peak body weight prior to decline until death. (**I**) Treatment with MSN-LEP-PIO extended disease duration in TDP-43 mice compared to controls. Transmission electron microscopy (TEM) images display MSN nanoparticle morphology and size distribution (**J**), while nitrogen adsorption-desorption isotherms characterize MSN, MSN-AP, and MSN-LEP-PIO samples. (**K**) Figures credit goes to Diana et al. under free access license 4.0 [41]

The mesoporous nanoparticle drug delivery system has an effective therapeutic approach for encapsulating hydrophilic and lipophilic drugs. Diana and his team recently developed a mesoporous silica nanoparticle bearing leptin and pioglitazone (MSN-LEP-PIO). The authors reported that an EDC coupling reaction chemically conjugated the mesoporous silica to drugs. His research findings suggested that MSN-LEP-PIO effectively controls motor function in TDP-43A315T mice. However, more evidence is still required at a molecular level to prove this hypothesis. Considering the facts, pioglitazone could be used to treat ALS through a nano platform. However, more evidence is required in contracts to maximize their potential to cross the BBB [129]. The details of experimental data are summarized in Fig. 6.

### Lipid nanoparticles

**Fig. 7:**
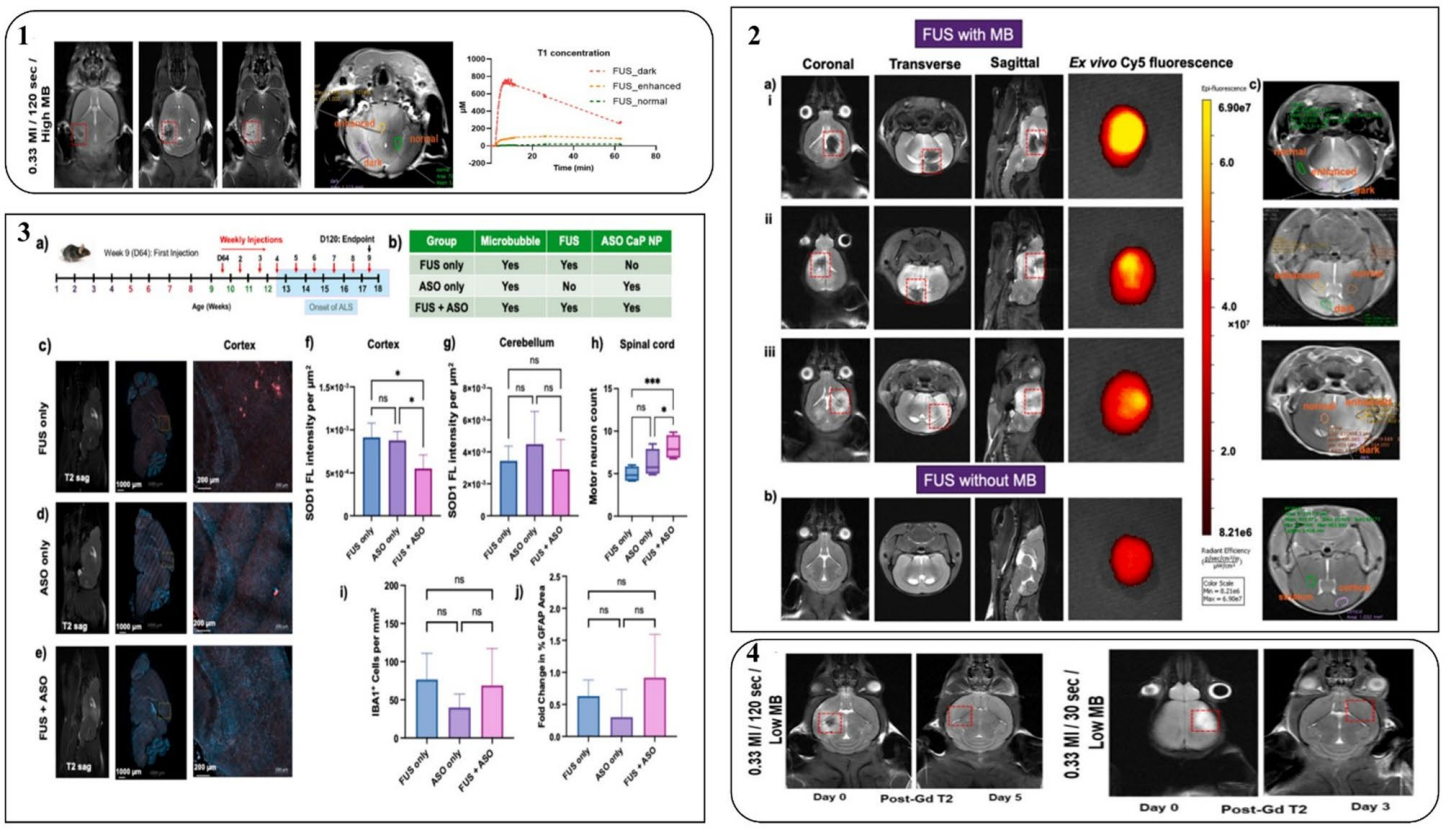
**1** Before and after gadolinium (Gd) administration, T2-weighted MRI scans (coronal view) and T2- and T1-weighted MRI scans show a marked disruption of the BBB, shown by a dotted square. In mice subjected to 0.33 MI/120 s/high microbubble dose, the T1-derived contrast substance concentration within the cortex exhibits the greatest up in the “dark” area, moderate uptake in the “enhanced” area, and negligible uptake in the “normal” region. **2** (a) T2-MRI pictures show ex vivo absorption of Cy5-SOD1 ASO Cap lipids NPs in the brains of mice administered focused ultrasound (FUS) and show BBB opening by Gd-based contrast enhancement (i, ii, iii correspond to three distinct mice from the same group). (b) Ex vivo fluorescence imaging confirms that T2-MRI scans from control animals exhibit low Cy5-SOD1 ASO Cap lipids NP uptake and no BBB opening. (c) To create graphs showing T1 and T2*-derived concentration variations within brain tissue, regions of interest were chosen, classified as cortical/striatum for the untreated group and dark/enhanced/normal for the FUS group. **3** (a) In G93A SOD1 mice, the experimental schedule describes recurrent weekly FUS administrations for BBB opening and therapeutic evaluation. (b) The experimental groups and parameters used in the therapy investigation are compiled in a table. (c–e) Representatives T2-weighted MRI images (left panels) and immunofluorescence staining for SOD1 (blue for Hoechst, red for human SOD1) indicate treatments with FUS only, ASO alone or combined FUS + ASO in the whole brain (middle panels) and the cerebral cortex regions (right panels). (f) A graph based on staining with anti-human SOD1 (ab13498) polyclonal antibodies demonstrates a considerably lower SOD1 level in the cortex of mice treated with FUS + ASO as compared to ASO only and FUS only controls. (g) Another graph shows that the cerebellum SOD1 levels in the FUS + ASO, ASO only, and FUS alone groups, as well as in the regions that FUS does not target (no = 3 FUS only, 4 ASO only, and 3 FUS + ASO), do not change significantly. (h) FUS + ASO-treated mice have considerably more motor neurons in their spinal cords than ASO-only and FUS-only controls, indicating that treatment is beneficial (*n* = 4 per group). Graphs show that there is no neuroinflammation after FUS therapy (*n* = 3 FUS only, 4 ASO only, 3 FUS + ASO), with no increase in microglial or astrocytic activation across FUS + ASO, ASO only, and FUS only groups. (h), where *p < 0.05; *p* < 0.01; *p < 0.001; and ns indicates no significant difference. The figure credit goes to corresponding authors under an open assessment license (http://creativecommons.org/licenses/by/4.0/) [130]

In order to be next, Gayathri et al. [130], demonstrated FUS lipid-based nanoparticles by delivering antisense oligonucleotides and tofersen to the murine brain for ALS therapy. This therapeutic approach offers a better outcome for reducing the burden of misfolded protein. Antisense oligonucleotides (ASOs) have the potential to silence proteins with gain-of-function mutations, such as SOD1, effectively. Therefore, the authors show that the next-generation SOD1 ASO (Tofersen) can be effectively delivered systemically into the brain of wild-type G93A-SOD1 transgenic C57BL/6 mice. Interestingly, they discover favourable results for BBB permeability and transitory without any indication of brain tissue injury, neuroinflammation, or structural alterations. It suggests that the suggested delivery method is secure and suitable for use in future translation studies [130]. However, more proof was needed to demonstrate these delivery methods’ safety and effectiveness profiles. The authors claimed that FUS-lipid nanoparticles are a promising noninvasive therapeutic strategy for the treatment of ALS. The details of preclinical results are summarized and discussed in Fig. 7.

### Liposomes

Liposomes are one of the oldest and most conventional nano-drug delivery carriers of active drugs targeting brain endothelial and astrocyte cells, maximizing local drug targeting [131, 132]. Yang and the groups demonstrated that verapamil is a well-known calcium channel blocker and potent P-gp inhibitor used experimentally to modulate drug resistance. By inhibiting P-gp, paclitaxel and andrographolide can significantly enhance CNS penetration of therapeutics normally refluxed by this transporter, including riluzole [133]. The research article presents a novel drug delivery platform for treating ALS. The drug was co-encapsulated in liposomes with riluzole and verapamil as a cocktail. Liposomes offer an efficient drug delivery vehicle due to their biocompatibility, ability to be targeted to specific tissues, and propensity to reduce systemic side effects. The co-encapsulation leverages verapamil’s ability to locally inhibit P-gp activity at the BBB and astrocyte interface, while riluzole exerts neuroprotective effects [134]. No promising liposomal formulation using Tofersen as an active drug is present in the literature. A few limitations are present with Tofersen, which are discussed just below.

## Concurrent regulatory updates

### Orphan drug

An orphan drug is a medication developed specifically to treat rare medical conditions, often called orphan diseases, which affect a small percentage of the population and would not be profitable to develop without government incentives and assistance. Despite their limited commercial viability, these drugs receive special status and regulatory support to encourage research and development. Tofersen is an orphan drug approved for treating ALS caused by mutations in the SOD1 gene, a rare genetic form of ALS [135]. The U.S. Food and Drug Administration (FDA) approved it on 25th Apr, 2023 [109, 136]. The drug is developed and marketed by Biogen, a biotechnology company known for its work in neurological diseases [137].

### U.S. Food and drug administration (US-FDA)

Under NDA no., the U.S. FDA granted accelerated approval on 25th Apr 2023. N215887, the proprietary name “QALSODY™” (tofersen) for the treatment of ALS associated with the SOD1 gene [138]. Qalsody is a prescription medication that Biogen MAInc develops. It is available in 100 mg/15Ml and administered intrathecal. It has been designated as a Reference Listed Drug (RLD) and a Reference Standard by the FDA [139]. This approval is based on reduced plasma NfL, a biomarker linked to neuronal damage in ALS [140]. Biogen is pursuing regulatory approval for tofersen through the FDA’s accelerated approval pathway, proposing the utilization of neurofilament as a surrogate biomarker that holds promise in predicting clinical benefits [141]. The below section deciphers the accelerated approval pathway and why tofersen was approved by accelerated pathways. The NDA encompassed findings derived from Phase 1 and Phase 1/2 trials conducted on healthy volunteers and data from the Phase 3 VALOR study and an open-label extension study.

Additionally, it incorporated 12-month integrated results from VALOR and the extension study. The 12-month analysis illustrated that the commencement of tofersen treatment at an earlier stage decelerated the rate of deterioration across various metrics of clinical strength and quality of life. Furthermore, it exhibited sustained decreases in neurofilament levels, indicating promising therapeutic efficacy in mitigating disease progression [142]. Tofersen was acquired by Biogen through a collaborative development and license agreement with Ionis Pharmaceuticals, permitting the conduct of clinical trials, regulatory endorsement, and commercialization. This agreement encompasses ongoing investigations, including the open-label extension study and the phase 3 ATLAS trial targeting presymptomatic individuals harbouring a SOD1 genetic mutation. The details of the clinical trials have been recorded in Table 4 [141].

### Accelerated approval of Tofersen

The FDA launched an accelerated approval program to enable earlier approval of medications that treat serious illnesses and address a medical need that remains based on a surrogate endpoint. A surrogate endpoint is a marker believed to predict clinical advantage but not a measure of clinical benefit itself. These include laboratory measurements, radiographic images, physical signs, and other measures. A surrogate endpoint can significantly reduce the time needed to obtain FDA approval [143]. Tofersen was approved under the FDA’s accelerated approval process on 25th Apr, 2023, because it treats a life-threatening condition—ALS caused by a mutation in the SOD1 gene for which there are yet no therapies [144].

### Health Canada

Health Canada granted marketing authorization with conditions (Notice of Compliance with Conditions (NOC/c)) for tofersen on 03rd Mar, 2025, for the treatment of adults with ALS associated with a mutation in the SOD1 gene [145]. It is only available by prescription and should be used under the supervision of healthcare professionals. Health Canada has accepted for review a new drug submission for tofersen for the treatment of a rare, genetic form of ALS, specifically SOD1-ALS, which represents approximately two per cent of all ALS cases. If sanctioned, Tofersen would represent a pioneering therapeutic approach in Canada specifically designed to address the genetic aetiology of ALS. The regulatory determination is anticipated to be rendered in the initial months of 2025 [146].

### European medicine agency (EMA)

The EMA is a responsible regulatory body that ensures the safety and efficacy of drugs before they are approved for use in the European Union. On 29th Aug, 2016, orphan designation (EU/3/16/1732) was granted by the European Commission to Biogen Idec Limited, United Kingdom, for synthetic ribonucleic acid oligonucleotide directed against superoxide dismutase one messenger ribonucleic acid (also known as BIIB067) for the treatment of ALS [147]. The European Commission has authorized Qalsody as the first treatment for SOD1-ALS, a rare genetic subtype affecting fewer than 1000 people in Europe [148]. The approval under exceptional circumstances reflects the urgent unmet medical need and the challenges of gathering large-scale clinical data for such a rare disease [149]. Given the limited treatment options for ALS during Qalsody evaluation, the EMA carefully weighed the medicine’s potential benefits against its known risks [150, 151]. Although the main clinical trial did not conclusively show that Qalsody slowed disease progression after 28 weeks, the EMA recognized that other supportive evidence confirmed the medicine’s expected mechanism of action and suggested a possible disease-modifying effect. Despite serious safety concerns, such as nervous system side effects, including spinal cord inflammation, the EMA judged these risks as manageable with appropriate medical care [151].

### Therapeutic goods administration (TGA)

The TGA reviewed and approved Tofersen, an orphan drug, on 05th Jan 2025, for use in Australia, with Biogen Australia Pty Ltd as the sponsor [152]. The approval process of tofersen involves the evaluation of the safety and efficacy of tofersen for the intended use in ALS linked to the SOD1 gene mutation. Tofersen is listed in the Australian Register of Therapeutic Goods (ARTG), allowing it to be legally supplied in the country [153].

## Future directions

There is a pressing need to identify reliable biomarkers for early detection and disease monitoring to enable timely interventions. Specifically, for tofersen, optimizing delivery methods to enhance CNS penetration and developing novel formulations to prolong its half-life are pivotal research areas [154]. Incorporating single-cell dynamics approaches could offer invaluable insights into the heterogeneity of neuronal responses to tofersen treatment, potentially revealing subpopulations of motor neurons with differential sensitivity or resistance [155]. Enhancing tofersen’s ability to reach affected motor neurons could significantly improve therapeutic efficacy, expand its clinical utility, and potentially offer more convenient dosing regimens for patients [156]. Tofersen’s potential as a nasal drug delivery system presents advantages such as non-invasiveness and direct brain targeting, particularly promising for SOD1 ALS [157]. Future efforts to enhance the delivery of tofersen to the brain could involve exploring strategies to increase its lipophilicity, facilitating its more effective crossing of the blood-brain barrier [158]. Techniques like lipid conjugation, nanoparticle formulations, prodrug design, and receptor-mediated transcytosis could be explored to modify tofersen’s properties and augment its ability to penetrate the BBB, thus amplifying therapeutic efficacy in conditions like SOD1-related ALS [159].

## Conclusion

The evolving therapeutic landscape for ALS is undergoing a significant transformation, which is marked by the landmark approval of Tofersen, the first gene-targeted therapy specially designed for patients with a mutation in the SOD1 gene. Tofersen was approved under the orphan drug category. Tofersen represents an important breakthrough in ALS treatment as a new direction, as the treatment shifted from conventional management to molecular precise therapies. Nanotherapeutics strategies have emerged as an innovative approach capable of revolutionizing CNS drug delivery in this category. Despite the promising results of Tofersen, the current route of administration (Intrathecal injection) poses significant clinical and logistical hurdles. It requires a direct injection into the spinal cord; this method, being invasive, leads to patient inconvenience and an increased burden on healthcare workers. Hence, there is a need to develop novel, noninvasive or less invasive delivery platforms with the aid of innovative nanocarriers and biocompatible materials. Simultaneously, the agencies such as the US FDA, EMA, TGA and Health Canada have taken progressive steps by granting accelerated approval and orphan status designation, reflecting a fast global commitment to fast-track the development of innovative ALS treatments.

## Data Availability

Data sharing is not applicable to this article as no new data were created or analysed in this study.

## Abbreviations

ALS: Amyotrophic lateral sclerosis
US-FDA: United States of Food and Drug Administration
SOD1: Superoxide dismutase 1
WHO: World Health Organization
NIH: National Institutes of Health
PK/PD: Pharmacokinetics and pharmacodynamics
S-ALS: Sporadic amyotrophic lateral sclerosis
F-ALS: Familial amyotrophic lateral sclerosis
TARDBP: Transactive response DNA binding protein
FDA: Food and Drug Administration
CNS: Central nervous system
BBB: Blood–brain barrier
NDDSs: Nanoparticle-based drug delivery systems
BSCB: Blood–spinal cord barrier
DMSO: Dimethyl sulfoxide
AgNPs: Silver nanoparticles
NfL: Neurofilament light chain
NfH: Neurofilament heavy chain
NDA: New drug application
pNfH: Phosphorylated neurofilament heavy chain
Tmax: Maximum plasma concentration
Cmax: Maximum observed concentration
t1/2: Terminal elimination half-life
FVC: Forced vital capacity
ALSFRS-R: ALS Functional Rating Scale-Revised
NIV: Noninvasive ventilation
ET: Essential tremor
FT: Fractional anisotropy

## References

[1] Kadena K, Vlamos P. Amyotrophic lateral sclerosis: current status in diagnostic biomarkers. Adv Exp Med Biol. 2020;1195:179–87. 32468476 10.1007/978-3-030-32633-3_26

[2] Mead RJ, Shan N, Reiser HJ, Marshall F, Shaw PJ. Amyotrophic lateral sclerosis: a neurodegenerative disorder poised for successful therapeutic translation. Nat Rev Drug Discov. 2023;22:185–212. 36543887 10.1038/s41573-022-00612-2 PMC9768794

[3] Miller TM, Cudkowicz ME, Genge A, Shaw PJ, Sobue G, Bucelli RC, et al. Trial of antisense oligonucleotide Tofersen for SOD1 ALS. N Engl J Med. 2022;387:1099–110. 36129998 10.1056/NEJMoa2204705

[4] Smith SE, McCoy-Gross K, Malcolm A, Oranski J, Markway JW, Miller TM, et al. Tofersen treatment leads to sustained stabilization of disease in SOD1 ALS in a real-world setting. Ann Clin Transl Neurol. 2025;12:311–9. 39783194 10.1002/acn3.52264 PMC11822806

[5] Mehta P, Raymond J, Zhang Y, Punjani R, Han M, Larson T et al. Prevalence of amyotrophic lateral sclerosis in the united States, 2018. Amyotroph Lateral Scler Frontotemporal Degener. 2023. p. 1–7. 10.1080/21678421.2023.2245858 37602649

[6] CDC. National Amyotrophic Lateral Sclerosis (ALS) Registry and Biorepository. 2020 ATSDR Annual Report; 2024. https://www.atsdr.cdc.gov/2020-annual-report/php/national-als-registry-and-biorepository.html. Accessed 15 Jul 2025.

[7] Mehta P, Raymond J, Nair T, Han M, Berry J, Punjani R, et al. Amyotrophic lateral sclerosis estimated prevalence cases from 2022 to 2030, data from the National ALS registry. Amyotroph Lateral Scler Frontotemporal Degener. 2025;26:290–5. 39749668 10.1080/21678421.2024.2447919

[8] Why this neurodegenerative disease needs. rare tag. The Times of India; 2023. https://timesofindia.indiatimes.com/city/delhi/why-this-neurodegenerative-disease-needs-rare-tag/articleshow/101176187.cms. Accessed 15 Jul 2025.

[9] Gendron TF, Petrucelli L. Immunological drivers of amyotrophic lateral sclerosis. Sci Transl Med. 2023;15:eadj9332. 37939160 10.1126/scitranslmed.adj9332

[10] Tzeplaeff L, Wilfling S, Requardt MV, Herdick M. Current state and future directions in the therapy of ALS. Cells. 2023;12:1523. 37296644 10.3390/cells12111523 PMC10252394

[11] Benatar M, Granit V, Andersen PM, Grignon A-L, McHutchison C, Cosentino S, et al. Mild motor impairment as prodromal state in amyotrophic lateral sclerosis: a new diagnostic entity. Brain. 2022;145:3500–8. 35594156 10.1093/brain/awac185 PMC9586537

[12] Bjelica B, Petri S. Narrative review of diagnosis, management, and treatment of dysphagia and sialorrhea in amyotrophic lateral sclerosis. J Neurol. 2024;271:6508–13. 39207520 10.1007/s00415-024-12657-x PMC11447084

[13] Masrori P, Van Damme P. Amyotrophic lateral sclerosis: a clinical review. Eur J Neurol. 2020;27:1918–29. 32526057 10.1111/ene.14393 PMC7540334

[14] Kaivola K, Pirinen M, Laaksovirta H, Jansson L, Rautila O, Launes J, et al. C9orf72 hexanucleotide repeat allele tagging snps: associations with ALS risk and longevity. Front Genet. 2023;14:1087098. 36936421 10.3389/fgene.2023.1087098 PMC10014923

[15] Van Daele SH, Moisse M, van Vugt JJFA, Zwamborn RAJ, van der Spek R, van Rheenen W, et al. Genetic variability in sporadic amyotrophic lateral sclerosis. Brain. 2023;146:3760–9. 37043475 10.1093/brain/awad120 PMC10473563

[16] Orange Book. Approved drug products with therapeutic equivalence evaluations. https://www.accessdata.fda.gov/scripts/cder/ob/results_product.cfm?Appl_Type=N&Appl_No=215887#42985.6. Accessed 15 July 2025.

[17] Orange Book. Approved drug products with therapeutic equivalence evaluations. https://www.accessdata.fda.gov/scripts/cder/ob/index.cfm#44884.6. Accessed 15 July 2025.

[18] Orange Book. Approved drug products with therapeutic equivalence evaluations. https://www.accessdata.fda.gov/scripts/cder/ob/results_product.cfm?Appl_Type=A&Appl_No=216035#44884.6. Accessed 15 July 2025.

[19] Qalsody. European Medicines Agency (EMA); 2024. https://www.ema.europa.eu/en/medicines/human/EPAR/qalsody. Accessed 15 July 2025.

[20] Brown RH, Al-Chalabi A. Amyotrophic lateral sclerosis. N Engl J Med. 2017;377:162–72. 28700839 10.1056/NEJMra1603471

[21] Taylor JP, Brown RH, Cleveland DW. Decoding ALS: from genes to mechanism. Nature. 2016;539:197–206. 27830784 10.1038/nature20413 PMC5585017

[22] Hardiman O, Al-Chalabi A, Chio A, Corr EM, Logroscino G, Robberecht W, et al. Amyotrophic lateral sclerosis. Nat Rev Dis Primers. 2017;3:17071. 28980624 10.1038/nrdp.2017.71

[23] Rai A, Shukla S, Gupta RK, Mishra A. ALS: a silent slayer of motor neurons. Traditional Chinese herbal medicine as an effective therapy. Curr Pharm Des. 2025;31:1328–46. 39835561 10.2174/0113816128329141241205063352

[24] PubChem. Riluzole. https://pubchem.ncbi.nlm.nih.gov/compound/5070. Accessed 10 July 2025.

[25] Search Orphan Drug Designations and Approvals. https://www.accessdata.fda.gov/scripts/opdlisting/oopd/detailedIndex.cfm?cfgridkey=072792. Accessed 24 July 2024.

[26] Radicava. (edaravone) FDA Approval History. Drugs.com. https://www.drugs.com/history/radicava.html. Accessed 28 July 2025.

[27] PubChem. Mci-186. https://pubchem.ncbi.nlm.nih.gov/compound/402. Accessed 27 July 2025.

[28] PubChem. Sodium phenylbutyrate/tauroursodeoxycholic acid. https://pubchem.ncbi.nlm.nih.gov/compound/12931649. Accessed 19 July 2025.

[29] Relyvrio ALZFORUM. https://www.alzforum.org/therapeutics/relyvrio. Accessed 19 July 2025.

[30] Amylyx Pharmaceuticals Announces FDA Approval of RELYVRIO™ for the Treatment of ALS, Amylyx. https://www.amylyx.com/news/amylyx-pharmaceuticals-announces-fda-approval-of-relyvriotm-for-the-treatment-of-als. Accessed 19 July 2025.

[31] PubChem. Tofersen. https://pubchem.ncbi.nlm.nih.gov/substance/507517373. Accessed 19 July 2025.

[32] Tofersen. First approval: drugs. 10.1007/s40265-023-01904-6. Accessed 19 July 2025.

[33] Pharma AL-S, Multicenter A, Label O. Single-Ascending Dose Study to Evaluate Safety, Tolerability, and Pharmacokinetics of AP-101 in Familial and Sporadic Amyotrophic Lateral Sclerosis (ALS). clinicaltrials.gov; 2020 Oct. Report No.: NCT03981536. https://clinicaltrials.gov/study/NCT03981536

[34] Ionis Pharmaceuticals. Inc. Phase 1–3 Study to Evaluate Efficacy, Safety, Pharmacokinetics and Pharmacodynamics of Intrathecally Administered ION363 in Amyotrophic Lateral Sclerosis Patients with Fused in Sarcoma Mutations (FUS-ALS). clinicaltrials.gov; 2024 Nov. Report No.: NCT04768972. https://clinicaltrials.gov/study/NCT04768972

[35] Shneider NA, Harms MB, Korobeynikov VA, Rifai OM, Hoover BN, Harrington EA, et al. Antisense oligonucleotide jacifusen for *FUS*-ALS: an investigator-initiated, multicentre, open-label case series. The Lancet. 2025;405:2075–86. 10.1016/S0140-6736(25)00513-6 PMC12407188 40414239

[36] Meyer T, Schumann P, Weydt P, Petri S, Koc Y, Spittel S, et al. Neurofilament light-chain response during therapy with antisense oligonucleotide Tofersen in *SOD1*-related ALS: treatment experience in clinical practice. Muscle Nerve. 2023;67:515–21. 36928619 10.1002/mus.27818

[37] Miller T, Cudkowicz M, Shaw PJ, Andersen PM, Atassi N, Bucelli RC, et al. Phase 1–2 trial of antisense oligonucleotide Tofersen for SOD1 ALS. N Engl J Med. 2020;383:109–19. 32640130 10.1056/NEJMoa2003715

[38] Tofersen (Qalsody). Use of a surrogate endpoint to demonstrate substantial evidence of effectiveness for an accelerated approval.

[39] Saini A, Chawla PA. Breaking barriers with tofersen: enhancing therapeutic opportunities in amyotrophic lateral sclerosis. Eur J Neurol. 2024;31:e16140. 37975798 10.1111/ene.16140 PMC11235929

[40] López-Pingarrón L, Almeida H, Soria-Aznar M, Reyes-Gonzales MC, Terrón MP, García JJ. Role of oxidative stress on the etiology and pathophysiology of amyotrophic lateral sclerosis (ALS) and its relationship with the enteric nervous system. Curr Issues Mol Biol. 2023;45:3315–32. 37185741 10.3390/cimb45040217 PMC10136958

[41] Obrador E, Salvador-Palmer R, López-Blanch R, Jihad-Jebbar A, Vallés SL, Estrela JM. The link between oxidative stress, redox status, bioenergetics and mitochondria in the pathophysiology of ALS. Int J Mol Sci. 2021;22:6352. 34198557 10.3390/ijms22126352 PMC8231819

[42] Peggion C, Scalcon V, Massimino ML, Nies K, Lopreiato R, Rigobello MP, et al. SOD1 in ALS: taking stock in pathogenic mechanisms and the role of glial and muscle cells. Antioxidants. 2022;11:614. 35453299 10.3390/antiox11040614 PMC9032988

[43] Wang H, Guan L, Deng M. Recent progress of the genetics of amyotrophic lateral sclerosis and challenges of gene therapy. Front Neurosci. 2023;17:1170996. 37250416 10.3389/fnins.2023.1170996 PMC10213321

[44] Berdyński M, Miszta P, Safranow K, Andersen PM, Morita M, Filipek S, et al. SOD1 mutations associated with amyotrophic lateral sclerosis analysis of variant severity. Sci Rep. 2022;12:103. 34996976 10.1038/s41598-021-03891-8 PMC8742055

[45] Collins M, Bowser R. Chapter 4—Molecular mechanisms of amyotrophic lateral sclerosis. In: Boulis N, O’Connor D, Donsante A, editors. Molecular and cellular therapies for motor neuron diseases. Academic Press; 2017. p. 61–99.

[46] DeJesus-Hernandez M, Mackenzie IR, Boeve BF, Boxer AL, Baker M, Rutherford NJ, et al. Expanded GGGGCC hexanucleotide repeat in noncoding region of C9ORF72 causes chromosome 9p-linked FTD and ALS. Neuron. 2011;72:245–56. 21944778 10.1016/j.neuron.2011.09.011 PMC3202986

[47] Renton AE, Majounie E, Waite A, Simón-Sánchez J, Rollinson S, Gibbs JR, et al. A hexanucleotide repeat expansion in C9ORF72 is the cause of chromosome 9p21-linked ALS-FTD. Neuron. 2011;72:257–68. 21944779 10.1016/j.neuron.2011.09.010 PMC3200438

[48] Ichiyanagi N, Fujimori K, Yano M, Ishihara-Fujisaki C, Sone T, Akiyama T, et al. Establishment of in vitro FUS-associated familial amyotrophic lateral sclerosis model using human induced pluripotent stem cells. Stem Cell Rep. 2016;6:496–510. 10.1016/j.stemcr.2016.02.011 PMC4834049 26997647

[49] Patel A, Lee HO, Jawerth L, Maharana S, Jahnel M, Hein MY, et al. A liquid-to-solid phase transition of the ALS protein FUS accelerated by disease mutation. Cell. 2015;162:1066–77. 26317470 10.1016/j.cell.2015.07.047

[50] Sau D, De Biasi S, Vitellaro-Zuccarello L, Riso P, Guarnieri S, Porrini M, et al. Mutation of SOD1 in ALS: a gain of a loss of function. Hum Mol Genet. 2007;16:1604–18. 17504823 10.1093/hmg/ddm110

[51] Kaur SJ, McKeown SR, Rashid S. Mutant SOD1 mediated pathogenesis of amyotrophic lateral sclerosis. Gene. 2016;577:109–18. 26657039 10.1016/j.gene.2015.11.049

[52] Neumann M, Sampathu DM, Kwong LK, Truax AC, Micsenyi MC, Chou TT, et al. Ubiquitinated TDP-43 in frontotemporal lobar degeneration and amyotrophic lateral sclerosis. Science. 2006;314:130–3. 17023659 10.1126/science.1134108

[53] Arai T, Hasegawa M, Akiyama H, Ikeda K, Nonaka T, Mori H, et al. TDP-43 is a component of ubiquitin-positive tau-negative inclusions in frontotemporal lobar degeneration and amyotrophic lateral sclerosis. Biochem Biophys Res Commun. 2006;351:602–11. 17084815 10.1016/j.bbrc.2006.10.093

[54] Honda H, Hamasaki H, Wakamiya T, Koyama S, Suzuki SO, Fujii N, et al. Loss of hnRNPA1 in ALS spinal cord motor neurons with TDP-43-positive inclusions. Neuropathology. 2015;35:37–43. 25338872 10.1111/neup.12153

[55] van Rheenen W, Shatunov A, Dekker AM, McLaughlin RL, Diekstra FP, Pulit SL, et al. Genome-wide association analyses identify new risk variants and the genetic architecture of amyotrophic lateral sclerosis. Nat Genet. 2016;48:1043–8. 27455348 10.1038/ng.3622 PMC5556360

[56] Iyer S, Acharya KR, Subramanian V. Prediction of structural consequences for disease causing variants in C21orf2 protein using computational approaches. J Biomol Struct Dyn. 2019;37:465–80. 29343210 10.1080/07391102.2018.1429313

[57] Jiao B, Xiao T, Hou L, Gu X, Zhou Y, Zhou L, et al. High prevalence of CHCHD10 mutation in patients with frontotemporal dementia from China. Brain. 2016;139:e21. 10.1093/brain/awv367. 26719383 10.1093/brain/awv367

[58] Dols-Icardo O, Nebot I, Gorostidi A, Ortega-Cubero S, Hernández I, Rojas-García R, et al. Analysis of the CHCHD10 gene in patients with frontotemporal dementia and amyotrophic lateral sclerosis from Spain. Brain. 2015;138:e400. 26152333 10.1093/brain/awv175

[59] Bannwarth S, Ait-El-Mkadem S, Chaussenot A, Genin EC, Lacas-Gervais S, Fragaki K, et al. Mitochondrial origin for frontotemporal dementia and amyotrophic lateral sclerosis through CHCHD10 involvement. Brain. 2014;137:2329–45. 24934289 10.1093/brain/awu138 PMC4107737

[60] Kenna KP, Van Doormaal PT, Dekker AM, Ticozzi N, Kenna BJ, Diekstra FP, et al. NEK1 variants confer susceptibility to amyotrophic lateral sclerosis. Nat Genet. 2016;48:1037–42. 27455347 10.1038/ng.3626 PMC5560030

[61] Picchiarelli G, Dupuis L. Role of RNA Binding Proteins with prison-like domains in muscle and neuromuscular diseases. Cell Stress. 2020;4:76. 32292882 10.15698/cst2020.04.217 PMC7146060

[62] Kao CS, van Bruggen R, Kim JR, Chen XX, Chan C, Lee J. Selective neuronal degeneration in MATR3 S85C knock-in mouse model of early-stage ALS. Nat Commun. 2020;11:5304. 33082323 10.1038/s41467-020-18949-w PMC7576598

[63] Couthouis J, Hart MP, Shorter J, De Jesus-Hernandez M, Erion R, Oristano R. A yeast functional screen predicts new candidate ALS disease genes. Proc Natl Acad Sci USA. 2011;108:20881–90. 22065782 10.1073/pnas.1109434108 PMC3248518

[64] Kapeli K, Pratt GA, Vu AQ, Hutt KR, Martinez FJ, Sundararaman B. Distinct and shared functions of ALS-associated proteins TDP-43, FUS and TAF15 revealed by multisystem analyses. Nat Commun. 2016;7:12143. 27378374 10.1038/ncomms12143 PMC4935974

[65] Ticozzi N, Vance C, Leclerc AL, Keagle P, Glass JD, McKenna-Yasek D, et al. Mutational analysis reveals the FUS homolog TAF15 as a candidate gene for familial amyotrophic lateral sclerosis. Am J Med Genet Part B Neuropsychiatr Genet. 2011;156B:285–90. 10.1002/ajmg.b.31158 21438137

[66] Kapeli K, Martinez FJ, Yeo GW. Genetic mutations in RNA-binding proteins and their roles in ALS. Human Genet. 2017;136:1193–214. 28762175 10.1007/s00439-017-1830-7 PMC5602095

[67] Elden AC, Kim HJ, Hart MP, Chen-Plotkin AS, Johnson BS, et al. Ataxin-2 intermediate-length polyglutamine expansions are associated with increased risk for ALS. Nature. 2010;466:1069–75. 20740007 10.1038/nature09320 PMC2965417

[68] Jin K, Li W, Nagayama T, He X, Sinor AD, Chang J, et al. Expression of the RNA-binding protein TIAR is increased in neurons after ischemic cerebral injury. J Neurosci Res. 2000;59:767–74. 10700014 10.1002/(SICI)1097-4547(20000315)59:6<767::AID-JNR9>3.0.CO;2-K

[69] Ravanidis S, Kattan F-G, Doxakis E. Unraveling the pathways to neuronal homeostasis and disease: mechanistic insights into the role of RNA-binding proteins and associated factors. Int J Mol Sci. 2018;19:2280. 30081499 10.3390/ijms19082280 PMC6121432

[70] Cirulli ET, Lasseigne BN, Petrovski S, Sapp PC, Dion PA, Leblond CS, et al. Exome sequencing in amyotrophic lateral sclerosis identifies risk genes and pathways. Science. 2015;347:1436–41. 25700176 10.1126/science.aaa3650 PMC4437632

[71] Freischmidt A, Müller K, Ludolph AC, Weishaupt JH, Andersen PM. Association of mutations in TBK1 with sporadic and familial amyotrophic lateral sclerosis and frontotemporal dementia. JAMA Neurol. 2017;74:110–3. 27892983 10.1001/jamaneurol.2016.3712

[72] Smith BN, Ticozzi N, Fallini C, Gkazi AS, Topp S, Kenna KP, et al. Exome-wide rare variant analysis identifies *TUBA4A* mutations associated with Familial ALS. Neuron. 2014;84:324–31. 25374358 10.1016/j.neuron.2014.09.027 PMC4521390

[73] Williams KL, Topp S, Yang S, Smith B, Fifita JA, Warraich ST, et al. Ccnf mutations in amyotrophic lateral sclerosis and frontotemporal dementia. Nat Commun. 2016;7:11253. 27080313 10.1038/ncomms11253 PMC4835537

[74] Nicolas A, Kenna KP, Renton AE, Ticozzi N, Faghri F, Chia R, et al. Genome-wide analyses identify KIF5A as a novel ALS gene. Neuron. 2018;97:1267–88. 29566793 10.1016/j.neuron.2018.02.027 PMC5867896

[75] Nahm M, Lim SM, Kim Y-E, Park J, Noh M-Y, Lee S, et al. ANXA11 mutations in ALS cause dysregulation of calcium homeostasis and stress granule dynamics. Sci Transl Med. 2020;12:eaax3993. 33087501 10.1126/scitranslmed.aax3993

[76] Cooper-Knock J, Moll T, Ramesh T, Castelli L, Beer A, Robins H, et al. Mutations in the glycosyltransferase domain of GLT8D1 are associated with Familial amyotrophic lateral sclerosis. Cell Rep. 2019;26:2298–e23065. 30811981 10.1016/j.celrep.2019.02.006 PMC7003067

[77] Orlacchio A, Babalini C, Borreca A, Patrono C, Massa R, Basaran S, et al. Spatacsin mutations cause autosomal recessive juvenile amyotrophic lateral sclerosis. Brain. 2010;133:591–8. 20110243 10.1093/brain/awp325 PMC2822627

[78] Ricci C, Marzocchi C, Battistini S. Micrornas as biomarkers in amyotrophic lateral sclerosis. Cells. 2018;7:219. 30463376 10.3390/cells7110219 PMC6262636

[79] Waller R, Wyles M, Heath PR, Kazoka M, Wollff H, Shaw PJ, et al. Small RNA sequencing of sporadic amyotrophic lateral sclerosis cerebrospinal fluid reveals differentially expressed MiRNAs related to neural and glial activity. Front Neurosci. 2017;11:731. 29375285 10.3389/fnins.2017.00731 PMC5767269

[80] Koval ED, Shaner C, Zhang P, du Maine X, Fischer K, Tay J, et al. Method for widespread microrna-155 inhibition prolongs survival in ALS-model mice. Hum Mol Genet. 2013;22:4127–35. 23740943 10.1093/hmg/ddt261 PMC3781640

[81] Zhou F, Zhang C, Guan Y, Chen Y, Lu Q, Jie L, et al. Screening the expression characteristics of several miRNAs in G93A-SOD1 transgenic mouse: altered expression of miRNA-124 is associated with astrocyte differentiation by targeting Sox2 and Sox9. J Neurochem. 2018;145:51–67. 28960306 10.1111/jnc.14229

[82] Cunha C, Santos C, Gomes C, Fernandes A, Correia AM, Sebastião AM, et al. Downregulated glia interplay and increased miRNA-155 as promising markers to track ALS at an early stage. Mol Neurobiol. 2018;55:4207–24. 28612258 10.1007/s12035-017-0631-2

[83] Ravnik-Glavač M, Glavač D. Circulating RNAs as potential biomarkers in amyotrophic lateral sclerosis. Int J Mol Sci. 2020;21:1714. 32138249 10.3390/ijms21051714 PMC7084402

[84] Nishimoto Y, Nakagawa S, Hirose T, Okano HJ, Takao M, Shibata S, et al. The long non-coding RNA nuclear-enriched abundant transcript 1_2 induces paraspeckle formation in the motor neuron during the early phase of amyotrophic lateral sclerosis. Mol Brain. 2013;6:31. 23835137 10.1186/1756-6606-6-31 PMC3729541

[85] Gagliardi S, Zucca S, Pandini C, Diamanti L, Bordoni M, Sproviero D, et al. Long non-coding and coding RNAs characterization in peripheral blood mononuclear cells and spinal cord from amyotrophic lateral sclerosis patients. Sci Rep. 2018;8:2378. 29402919 10.1038/s41598-018-20679-5 PMC5799454

[86] Zhang F, Zhang Q, Ke Y, Hao J, Lu L, Lu N, et al. Serum uric acid levels in patients with amyotrophic lateral sclerosis: a meta-analysis. Sci Rep. 2018;8:1100. 29348425 10.1038/s41598-018-19609-2 PMC5773600

[87] Steinacker P, Verde F, Fang L, Feneberg E, Oeckl P, Roeber S, et al. Chitotriosidase (CHIT1) is increased in microglia and macrophages in spinal cord of amyotrophic lateral sclerosis and cerebrospinal fluid levels correlate with disease severity and progression. J Neurol Neurosurg Psychiatry. 2018;89:239–47. 29142138 10.1136/jnnp-2017-317138

[88] Verde F, Steinacker P, Weishaupt JH, Kassubek J, Oeckl P, Halbgebauer S, et al. Neurofilament light chain in serum for the diagnosis of amyotrophic lateral sclerosis. J Neurol Neurosurg Psychiatry. 2019;90:157–64. 30309882 10.1136/jnnp-2018-318704

[89] An efficacy, safety, tolerability, pharmacokinetics and pharmacodynamics study of biib067 in adults with inherited amyotrophic lateral sclerosis (ALS) (VALOR (Part C))—Mayo Clinic. https://www.mayo.edu/research/clinical-trials/cls-20468264. Accessed 16 July 2025.

[90] Trial. Long-Term Evaluation of BIIB067 (Tofersen). ALS Therapy Development Institute. https://www.als.net/als-trial-navigator/296/. Accessed 15 July 2025.

[91] Ly CV, Miller TM. Emerging antisense oligonucleotide and viral therapies for amyotrophic lateral sclerosis. Curr Opin Neurol. 2018;31:648–54. 30028737 10.1097/WCO.0000000000000594 PMC7291817

[92] Hoxhaj P, Hastings N, Kachhadia MP, Gupta R, Sindhu U, Durve SA, et al. Exploring advancements in the treatment of amyotrophic lateral sclerosis: a comprehensive review of current modalities and future prospects. Cureus. 2023;15:e45489. 37868386 10.7759/cureus.45489 PMC10585945

[93] Gianferrari G, Martinelli I, Simonini C, Zucchi E, Fini N, Carra S, et al. Case report: p. Glu134del SOD1 mutation in two apparently unrelated ALS patients with mirrored phenotype. Front Neurol. 2022;13:1052341. 36686515 10.3389/fneur.2022.1052341 PMC9846158

[94] Vidovic M, Menschikowski M, Freigang M, Lapp HS, Günther R. Macrophage inclusions in cerebrospinal fluid following treatment initiation with antisense oligonucleotide therapies in motor neuron diseases. Neurol Res Pract. 2024;6:11. 38383503 10.1186/s42466-023-00305-0 PMC10882918

[95] Tofersen. https://go.drugbank.com/drugs/DB14782. Accessed 15 July 2025.

[96] Cerillo JL, Parmar M. Tofersen. StatPearls. Treasure Island: StatPearls Publishing; 2025. 37603661

[97] Research C, for DE and. FDA approves treatment of amyotrophic lateral sclerosis associated with a mutation in the SOD1 gene. FDA; 2024. https://www.fda.gov/drugs/news-events-human-drugs/fda-approves-treatment-amyotrophic-lateral-sclerosis-associated-mutation-sod1-gene. Accessed 15 July 2025.

[98] Biogen A. Study to evaluate efficacy, safety, tolerability, pharmacokinetics, and pharmacodynamics of BIIB067 administered to adult subjects with amyotrophic lateral sclerosis and confirmed superoxide dismutase 1 mutation. clinicaltrials.gov; 2023. Report No.: NCT02623699. https://clinicaltrials.gov/study/NCT02623699

[99] Tofersen Monograph for Professionals. Drugs.com. https://www.drugs.com/monograph/tofersen.html. Accessed 15 July 2025.

[100] Lovett A, Chary S, Babu S, Bruneteau G, Glass JD, Karlsborg M, et al. Serious neurologic adverse events in Tofersen clinical trials for amyotrophic lateral sclerosis. Muscle Nerve. 2025;71:1006–15. 40017137 10.1002/mus.28372 PMC12060635

[101] Shaw P, Miller T, Cudkowicz M, Genge A, Sobue G, Nestorov I, et al. Tofersen in adults with SOD1-ALS: phase 3 VALOR trial and open-label extension results. J Neurol Neurosurg Psychiatry. 2022;93:e2.

[102] Long-term treatment of SOD1 ALS. with Tofersen: a multicentre experience in 17 patients. J Neurol. https://link.springer.com/article/10.1007/s00415-024-12437-7 10.1007/s00415-024-12437-7 38829431

[103] Tofersen decreases neurofilament levels. supporting the pathogenesis of the SOD1 p.D91A variant in amyotrophic lateral sclerosis patients: Communications Medicine. https://www.nature.com/articles/s43856-024-00573 10.1038/s43856-024-00573-0 PMC11272917 39054363

[104] Neurodegenerative. and neuroinflammatory changes in SOD1-ALS patients receiving Tofersen: Scientific Reports. https://www.nature.com/articles/s41598-025-94984-1. Accessed 23 July 2025. 10.1038/s41598-025-94984-1 PMC11961715 40169784

[105] Newman J, Corcia P, Genge A, Bucelli RC. SOD1-ALS from gene discovery to targeted therapeutics: a comprehensive review; 2025. https://touchneurology.com/brain-trauma/journal-articles/sod1-als-from-gene-discovery-to-targeted-therapeutics-a-comprehensive-review/

[106] Oliveira Santos M, de Carvalho M. Profiling tofersen as a treatment of superoxide dismutase 1 amyotrophic lateral sclerosis. Expert Rev Neurother. 2024;24:549–53. 10.1080/14737175.2024.2355983. 38758193 10.1080/14737175.2024.2355983

[107] FDA accelerated approval of anticancer agents. J Clin Oncol. 10.1200/jco.2010.28.15_suppl.6065

[108] Capitalizing on Hope: questionable marketing approval and pricing of a new ALS drug—Matthew B. Flynn, James F. Flynn, Ana M. Palacios; 2024. https://journals.sagepub.com/doi/10.1177/27551938241247778 10.1177/27551938241247778 38646691

[109] Tofersen. (Qalsody) for ALS: the Medical Letter Inc. https://secure.medicalletter.org/TML-article-1681a. Accessed 19 July 2025.

[110] Miller RG, Bouchard JP, Duquette P, Eisen A, Gelinas D, Harati Y, et al. Clinical trials of riluzole in patients with ALS. Neurology. 1996;47:S86-92. 8858057 10.1212/wnl.47.4_suppl_2.86s

[111] Smith SE, Miller TM. Antisense oligonucleotides for amyotrophic lateral sclerosis. In: Tuszynski MH, editor. Translational neuroscience: fundamental approaches for neurological disorders. Cham: Springer Nature Switzerland; 2025. pp. p185–200.

[112] FDA approves treatment. of amyotrophic lateral sclerosis associated with a mutation in the SOD1 gene: FDA. https://www.fda.gov/drugs/news-events-human-drugs/fda-approves-treatment-amyotrophic-lateral-sclerosis-associated-mutation-sod1-gene. Accessed 18 Jul 2025.

[113] Biogen, Ionis Announce Discontinuation of BIIB078 in C9orf72-Associated ALS. Neurology live; 2022. https://www.neurologylive.com/view/biogen-ionis-discontine-biib078-c9orf72-associated-amyotrophic-lateral-sclerosis. Accessed 23 July 2025.

[114] Biogen A. Phase 1 multiple-ascending-dose study to assess the safety, tolerability, and pharmacokinetics of BIIB078 administered intrathecally to adults with C9ORF72-associated amyotrophic lateral sclerosis. clinicaltrials.gov; 2022 Jan. Report No.: NCT03626012. https://clinicaltrials.gov/study/NCT03626012. Accessed 15 July 2025.

[115] Wave Life Sciences Ltd, Multicenter A. Randomized, Double-blind, Placebo-controlled, Phase 1b/2a Study of WVE-004 Administered Intrathecally to Patients with C9orf72-associated Amyotrophic Lateral Sclerosis (ALS) or Frontotemporal Dementia (FTD). clinicaltrials.gov; 2023 Oct. Report No.: NCT04931862. https://clinicaltrials.gov/study/NCT04931862. Accessed 26 July 2025.

[116] Intellia Announces First Clinical Evidence from Ongoing Phase 1. Study that Nexiguran Ziclumeran (nex-z), an In Vivo CRISPR/Cas9-Based Gene Editing Therapy, May Favorably Impact Disease Progression in Transthyretin (ATTR) Amyloidosis - Intellia Therapeutics. https://ir.intelliatx.com/news-releases/news-release-details/intellia-announces-first-clinical-evidence-ongoing-phase-1-study. Accessed 23 Jul 2025.

[117] Deng H-X, Zhai H, Shi Y, Liu G, Lowry J, Liu B, et al. Efficacy and long-term safety of CRISPR/Cas9 genome editing in the SOD1-linked mouse models of ALS. Commun Biol. 2021;4:396. 33767386 10.1038/s42003-021-01942-4 PMC7994668

[118] DeVos SL, Miller TM. Direct intraventricular delivery of drugs to the rodent central nervous system. J Vis Exp. 2013;75:50326. 10.3791/50326 PMC3679837 23712122

[119] Wang GY, Rayner SL, Chung R, Shi BY, Liang XJ. Advances in nanotechnology-based strategies for the treatments of amyotrophic lateral sclerosis. Mater Today Bio. 2020;6:100055. 32529183 10.1016/j.mtbio.2020.100055 PMC7280770

[120] Nabizadeh F. Biomaterials in the treatment of amyotrophic lateral sclerosis. Neurology Letters. 2022;1:12–6.

[121] Sharma N, Kumar A, Sambhakar S, Bhatia D, Hussain S, Mursal M, et al. Recent advances in nanotherapeutics and theranostics for squamous cell carcinoma: a comprehensive review. Current Drug Deliv. 2025;22:1–19. 10.2174/0115672018342513241230061704 39865830

[122] Kumar V, Nair SC. Nano lipid carriers as a promising drug delivery carrier for neurodegenerative disorders - an overview of recent advances. Recent Pat Biotechnol. 2024;18(1):2–21. 38205772 10.2174/1872208317666230320164219

[123] Mittal KR, Pharasi N, Sarna B, Singh M, Rachana null, Haider S, et al. Nanotechnology-based drug delivery for the treatment of CNS disorders. Transl Neurosci. 2022;13:527–46. 36741545 10.1515/tnsci-2022-0258 PMC9883694

[124] Ilić T, Đoković JB, Nikolić I, Mitrović JR, Pantelić I, Savić SD, et al. Parenteral Lipid-Based nanoparticles for CNS disorders: integrating various facets of preclinical evaluation towards more effective clinical translation. Pharmaceutics. 2023;15:443. 36839768 10.3390/pharmaceutics15020443 PMC9966342

[125] Srivastava S, Kumar A, Yadav P, Kumar, Mathew J, Chourasia M. Formulation and performance evaluation of polymeric mixed micelles encapsulated with Baicalein for breast cancer treatment. Drug Dev Ind Pharm. 2021;47:1–38. 34781796 10.1080/03639045.2021.2007394

[126] Maurya P, Saklani R, Singh S, Nisha R, Mishra N, Singh P, et al. Effective uptake of folate-functionalized ethionamide-loaded hybrid system: targeting alveolar macrophages. Nanomedicine. 2022;17:1819–31. 36136373 10.2217/nnm-2021-0468

[127] Kumar A, Dhiman C, Narayan KP, Kumar M, Sharma M, Nanotherapeutics: restoring gut-dysbiosis and inducing cancer apoptosis. Spatially variable genes in cancer: development, progression, and treatment response. IGI Global Scientific Publishing; 2025. pp. 177–204. https://www.igi-global.com/chapter/nanotherapeutics/www.igi-global.com/chapter/nanotherapeutics/366020. Accessed 15 July 2025.

[128] Jojo GM, Kuppusamy G. Scope of new formulation approaches in the repurposing of Pioglitazone for the management of Alzheimer’s disease. J Clin Pharm Ther. 2019;44:337–48. 30738020 10.1111/jcpt.12808

[129] Díaz-García D, Ferrer-Donato Á, Méndez-Arriaga JM, Cabrera-Pinto M, Díaz-Sánchez M, Prashar S, et al. Design of mesoporous silica nanoparticles for the treatment of amyotrophic lateral sclerosis (ALS) with a therapeutic cocktail based on leptin and pioglitazone. ACS Biomater Sci Eng. 2022;8:4838–49. 36240025 10.1021/acsbiomaterials.2c00865 PMC9667463

[130] Ediriweera GR, Sivaram AJ, Cowin G, Brown ML, McAlary L, Lum JS, et al. Lipid nanoparticles and transcranial focused ultrasound enhance the delivery of SOD1 antisense oligonucleotides to the murine brain for ALS therapy. J Control Release. 2025;378:221–35. 39645085 10.1016/j.jconrel.2024.11.074

[131] Sharma M, Joshi J, Chouhan NK, Talati MN, Vaidya S, Kumar A, et al. Liposome-a comprehensive approach for researchers. Molecular pharmacology. IntechOpen; 2020.

[132] Kumar A, Rana R, Saklani R, Kumar M, Yadav PK, Tiwari A, et al. Technology transfer of a validated RP-HPLC method for the simultaneous estimation of Andrographolide and Paclitaxel in application to pharmaceutical nanoformulation. J Chromatogr Sci. 2024;62:356–63. 37674403 10.1093/chromsci/bmad070

[133] Singh R, Kumar A, Srivastava D, Narayan KP, Chaurasia M, Flora SJS. Nano-formulation technology for simultaneous Paclitaxel and Andrographolide delivery: pre-formulation insights. Essential Chem. 2025;2:2533990.

[134] Yang T, Ferrill L, Gallant L, McGillicuddy S, Fernandes T, Schields N, et al. Verapamil and riluzole cocktail liposomes overcome pharmacoresistance by inhibiting P-glycoprotein in brain endothelial and astrocyte cells: a potent approach to treat amyotrophic lateral sclerosis. Eur J Pharm Sci. 2018;120:30–9. 29704642 10.1016/j.ejps.2018.04.026

[135] Tofersen. The ALS Association. https://www.als.org/navigating-als/living-with-als/fda-approved-drugs/tofersen. Accessed 15 July 2025.

[136] Orange Book. Approved Drug Products with Therapeutic Equivalence Evaluations. https://www.accessdata.fda.gov/scripts/cder/ob/results_product.cfm?Appl_Type=N&Appl_No=215887#42909. Accessed 15 July 2025.

[137] Jin J, Zhong X-B. ASO drug Qalsody (tofersen) targets amyotrophic lateral sclerosis. Trends Pharmacol Sci. 2023;44:1043–4. 37709589 10.1016/j.tips.2023.08.008 PMC10841252

[138] van Roon-Mom W, Ferguson C, Aartsma-Rus A. From failure to meet the clinical endpoint to U.S. food and drug administration approval: 15th antisense oligonucleotide therapy approved Qalsody (Tofersen) for treatment of SOD1 mutated amyotrophic lateral sclerosis. Nucleic Acid Ther. 2023;33:234–7. 37581487 10.1089/nat.2023.0027

[139] Research C for DE and. Drug Trials Snapshots: QALSODY; 2024. https://www.fda.gov/drugs/drug-approvals-and-databases/drug-trials-snapshots-qalsody. Accessed 15 July 2025.

[140] FDA approves QALSODY. ^™^ (tofersen) as the first treatment targeting a genetic cause of ALS: Ionis Pharmaceuticals, Inc. https://ir.ionis.com/news-releases/news-release-details/fda-approves-qalsodytm-tofersen-first-treatment-targeting. Accessed 15 July 2025.

[141] FDA Grants Accelerated Approval. for QALSODY^™^ (tofersen) for SOD1-ALS, a Major Scientific Advancement as the First Treatment to Target a Genetic Cause of ALS: Biogen. https://investors.biogen.com/news-releases/news-release-details/fda-grants-accelerated-approval-qalsodytm-tofersen-sod1-als. Accessed 16 Jul 2025.

[142] Wiesenfarth M, Dorst J, Brenner D, Elmas Z, Parlak Ö, Uzelac Z, et al. Effects of Tofersen treatment in patients with SOD1-ALS in a real-world setting - a 12-month multicenter cohort study from the German early access program. EClinicalMedicine. 2024;69:102495. 38384337 10.1016/j.eclinm.2024.102495 PMC10878861

[143] Research C for DE and. Accelerated Approval Program. FDA. FDA; 2024. https://www.fda.gov/drugs/nda-and-bla-approvals/accelerated-approval-program. Accessed 15 July 2025.

[144] FDA approves first ALS treatment via accelerated approval. The ALS Association. https://www.als.org/stories-news/fda-approves-first-als-treatment-accelerated-approval. Accessed 15 July 2025.

[145] Brown CA, Lally C, Kupelian V, Flanders WD. Estimated prevalence and incidence of amyotrophic lateral sclerosis and SOD1 and C9orf72 genetic variants. Neuroepidemiology. 2021;55:342–53. 34247168 10.1159/000516752

[146] Health Canada accepts for review new drug submission for Tofersen for treatment of rare. genetic form of ALS. https://www.biogen.ca/en-ca/news/2024-03-11-news.html. Accessed 15 July 2025.

[147] EU/3/16/1732—orphan designation for treatment of amyotrophic lateral sclerosis: European Medicines Agency (EMA). 2016. https://www.ema.europa.eu/en/medicines/human/orphan-designations/eu-3-16-1732. Accessed 16 July 2025.

[148] EU Approves Biogen’s Tofersen as First Treatment for SOD1 ALS. Neurology live; 2024. https://www.neurologylive.com/view/eu-approves-biogen-tofersen-first-treatment-sod1-als. Accessed 15 July 2025.

[149] New treatment for rare motor neurone disease recommended for approval: European Medicines Agency (EMA); 2024. https://www.ema.europa.eu/en/news/new-treatment-rare-motor-neurone-disease-recommended-approval. Accessed 10 July 2025.

[150] European Medicines Agency Accepts Tofersen Marketing Authorization Application to Treat Rare. Genetic form of ALS: Biogen. https://investors.biogen.com/news-releases/news-release-details/european-medicines-agency-accepts-tofersen-marketing. Accessed 10 July 2025.

[151] Biogen’s QALSODY^®^ (tofersen.), the First Therapy to Treat Rare, Genetic Form of ALS, Received Positive Opinion from CHMP: Biogen. https://investors.biogen.com/news-releases/news-release-details/biogens-qalsodyr-tofersen-first-therapy-treat-rare-genetic-form. Accessed 16 July 2025.

[152] Administration (TGA) TG. Notice for tofersen (Biogen Australia Pty Ltd): Therapeutic Goods Administration (TGA). Therapeutic Goods Administration (TGA); 2025. https://www.tga.gov.au/resources/designations-determinations/notice-tofersen-biogen-australia-pty-ltd. Accessed 16 July 2025.

[153] Administration (TGA) TG. QALSODY Biogen Australia Pty Ltd: Therapeutic Goods Administration (TGA). Therapeutic Goods Administration (TGA). 2025. https://www.tga.gov.au/resources/prescription-medicines-under-evaluation/qalsody-biogen-australia-pty-ltd. Accessed 16 Jul 2025.

[154] Doroszkiewicz J, Groblewska M, Mroczko B. Molecular biomarkers and their implications for the early diagnosis of selected neurodegenerative diseases. Int J Mol Sci. 2022;23:4610. 35563001 10.3390/ijms23094610 PMC9100918

[155] Sant P, Rippe K, Mallm J-P. Approaches for single-cell RNA sequencing across tissues and cell types. Transcription. 2023;14:127–45. 37062951 10.1080/21541264.2023.2200721 PMC10807473

[156] Cudkowicz M, Miller T, Shaw P, Bennett CF, Lane R, Chang I, et al. Tofersen, a SOD1 antisense oligonucleotide in participants with ALS—results from a multiple dose study. Neurology. 2020;94:657.

[157] Shukla S, Tiwari S, Bhattacharya R, Ojha S, Mishra S, Gupta SK. Nanotechnology-based approaches for nose-to-brain drug delivery in neurodegenerative diseases. Lett Drug Des Discovery. 2024;21:1913–21.

[158] Wu D, Chen Q, Chen X, Han F, Chen Z, Wang Y. The blood-brain barrier: structure, regulation, and drug delivery. Signal Transduct Target Ther. 2023;8:217. 37231000 10.1038/s41392-023-01481-w PMC10212980

[159] Shilpi S, Jain A, Dixit S, Saraogi G, Yadav AK, Jain SK. Chapter 4—Lipid nanocarrier-based drug delivery for the treatment of brain-related disorders. In: Yadav AK, Shukla R, Flora SJS, editors. Nanomedical drug delivery for neurodegenerative diseases. Academic; 2022. p. 55–65.

